# A red-shifted two-photon-only caging group for three-dimensional photorelease†
†Electronic supplementary information (ESI) available. See DOI: 10.1039/c7sc05182d


**DOI:** 10.1039/c7sc05182d

**Published:** 2018-02-09

**Authors:** Yvonne Becker, Erik Unger, Manuela A. H. Fichte, Daniel A. Gacek, Andreas Dreuw, Josef Wachtveitl, Peter J. Walla, Alexander Heckel

**Affiliations:** a Goethe University Frankfurt , Institute for Organic Chemistry and Chemical Biology , Max-von-Laue-Str. 7 , 60438 Frankfurt , Germany . Email: heckel@uni-frankfurt.de; b Technical University Braunschweig , Institute for Physical and Theoretical Chemistry , Gaußstr. 17 , 38106 Braunschweig , Germany; c Interdisciplinary Center for Scientific Computing (IWR) , Theoretical and Computational Chemistry , Im Neuenheimer Feld 205A , 69120 Heidelberg , Germany; d Goethe University Frankfurt , Institute for Physical and Theoretical Chemistry , Max-von-Laue-Str. 7 , 60438 Frankfurt , Germany

## Abstract

Based on nitrodibenzofuran (NDBF) a new photocage with higher two-photon action cross section and red-shifted absorption was developed. Due to calculations, a dimethylamino functionality (DMA) was added at ring position 7. The uncaging of nucleobases after two-photon excitation (2PE) could be visualized *via* double-strand displacement in a hydrogel. With this assay we achieved three-dimensional photorelease of DMA-NDBF-protected DNA orthogonal to NDBF-protected strands. While being an excellent 2P-cage, DMA-NDBF is surprisingly stable under visible-light one-photon excitation (1PE). This case of excitation-specific photochemistry enhances the scope of orthogonal photoregulation.

## Abstract 2

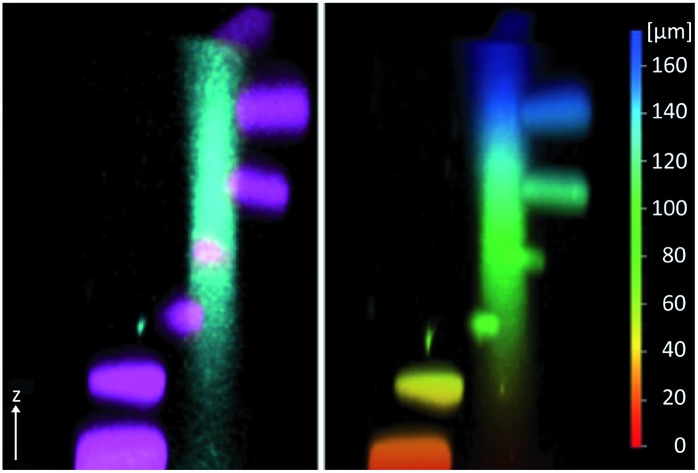
With a new photolabile protecting group – exclusively cleavable by two-photon-excitation – complex light scenarios for three-dimensional uncaging are possible.

## Introduction

Over the last decades photolabile protecting groups (PPGs) became a frequently used tool to regulate bioactive molecules^1^ such as neurotransmitters,^2^–^5^ hormones^6^,^7^ and even macro-molecules like proteins^8^,^9^ and oligonucleotides.^10^–^12^

One crucial long-term goal is a red-shifting^13^ of the light-induced photorelease (uncaging^14^) into the *therapeutic window* (∼650–950 nm (ref. 15)). It is less harmless for living cells and deeper tissue penetration becomes possible in biological applications due to less absorption and scattering of *e.g.* blood.^16^ In the 1990s a promising (un)caging strategy for higher wavelengths (>650 nm) has emerged, based on two-photon (2P) sensitive photolabile groups.^17^–^20^ PPGs with 2P-absorption character are cleavable with femtosecond pulsed lasers. This non-linear optical process can be seen as a simultaneous absorption of two photons. In many cases – but not all^21^ – the resulting electronically excited state is the same when photons of half the energy are used. This process was first described by Maria Göppert-Mayer.^22^ It can be used to realize photochemistry with 3D spatial resolution since the excitation depends on the squared intensity (*p* ∼ *I*^2^). Excitation volumes can be as small as a femtoliter.^16^ The 2P-uncaging efficiency *δ*_u_ can be described (analogous to the “quantum product” in 1P-uncaging) by the product of absorption cross section *δ*_a_ and the uncaging quantum yield *Φ*_u_ (*δ*_u_ = *δ*_a_·*Φ*_u_).^16^ The *δ*_a_ of chromophores depends on the length and planarity of the π-electron system and substituent effects (*i.e.* push–pull-systems).^16^,^23^,^24^ The positions of the substituents are crucial: a dipolar character as well as quadrupolar or octupolar enhance the 2P-absorption.^23^

In 2006, Ellis-Davies *et al.* introduced the new chromophore 3-nitrodibenzofuran (NDBF) for 2P-photolysis of NDBF-EGTA:Ca^2+^ with *δ*_u_ = 0.6 GM at 720 nm.^25^

**Fig. 1:**
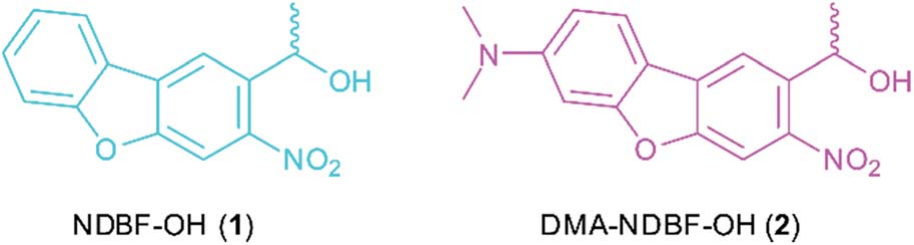
The caging group precursor NDBF-OH (**1**) and its new dimethylamino derivative DMA-NDBF-OH (**2**).

In this paper, based on the NDBF core (see compound **1**, Fig. 1), we rationally designed and synthesized the dimethylamino derivate DMA-NDBF-OH (**2**, Fig. 1). Due to calculations with the DFT/B3LYP method and a 6-31*G basis set for the ground state equilibrium structures and TDDFT/BHLYP for exited states the dipolar structure should be red-shifted and have an increased *δ*_a_.^26^ Despite the availability of sophisticated computational methods the optimisation of 2P-chromophors remains a formidable challenge.

### Experimental and results

The simulation of various NDBF derivatives predicted a preferred substitution of ring-position 7 with a dimethylamino (DMA) functionality as donor (Fig. 1). The expected uncaging efficiency of DMA-NDBF based on computed 2P-absorption spectra should be more than 20 times higher than the one of NDBF at its respective red-shifted maximum.^26^

### Small molecule synthesis and characterisation

**Scheme 1:**
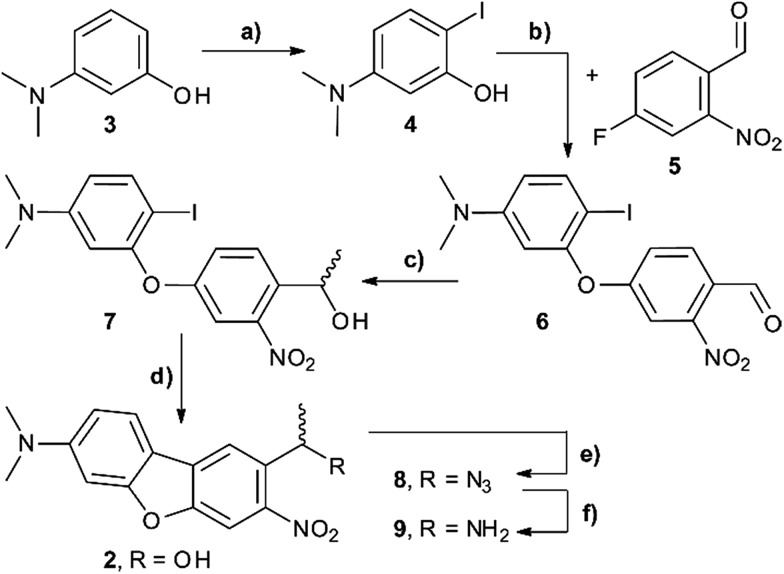
Synthesis of photocage **9**. (a) KI, KIO_3_, 1 N H_2_SO_4_, H_2_O, 3 h, rt, 52%. (b) KO*t*Bu, DMSO, 63%. (c) Al(CH_3_)_3_, CH_2_Cl_2_, 30 min, 0 °C, 97%. (d) Cs_2_CO_3_, Pd(OAc)_2_, H_2_O, DMAc, 3 d, 80 °C, 49%. (e) PPh_3_, HN_3_ (*in situ*), DEAD, THF 12 h, 0 °C – > rt, 93%. (f) PPh_3_, H_2_O, THF/MeCN (1 : 1 v/v), 20 h, 70 °C – > rt, 71%. DMAc = dimethylacetamide, DEAD = diethyl azodicarboxylate.

The synthesis of the caging group precursor DMA-NDBF-NH_2_ (**9**) started with the iodation of 3-dimethyl-aminophenol (**3**) using KI and KIO_3_, followed by an electrophilic aromatic substitution to form 2-iodo-5-dimethylaminophenol (**4**) as summarised in Scheme 1. The unsymmetrical aryl ether **6** was formed *via* coupling with 4-fluoro-2-nitrobenzaldehyde (**5**) and subsequently reduced with trimethylaluminum. Hydrolysis led to alcohol **7**, which was used for a palladium-catalysed intramolecular Heck-like reaction to yield the closed-ring form **2**. The azide **8** was synthesised under Mitsunobu conditions with *in situ* generated HN_3_. 8-(1-Aminoethyl)-*N*,*N*-dimethyl-7-nitrodibenzo-[*b*,*d*]furan-3-amine **9** was finally obtained *via* Staudinger reaction.

**Fig. 2:**
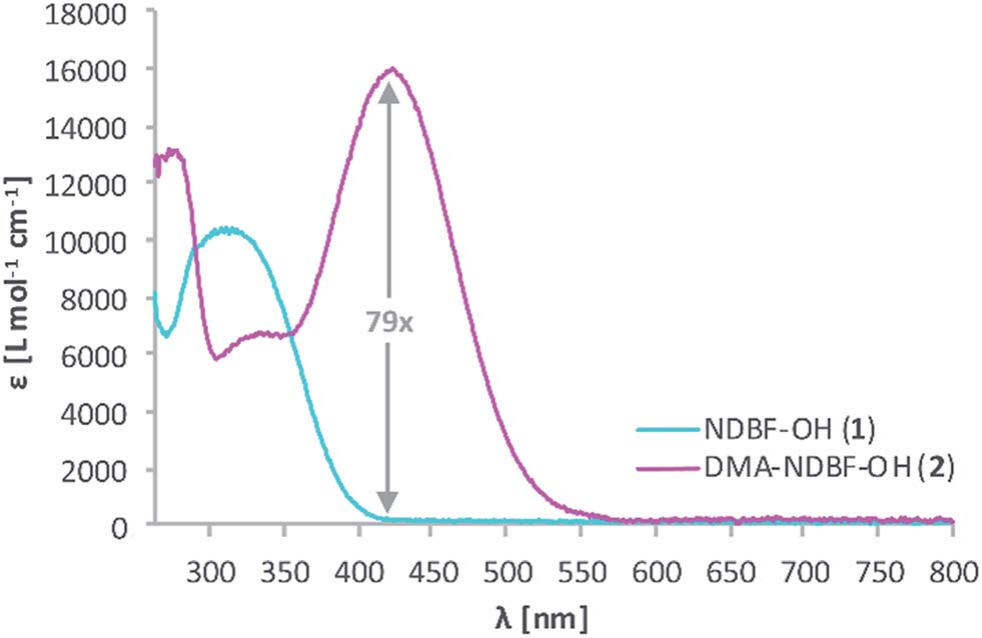
Comparison of the 1P-absorption spectra of NDBF-OH (**1**, cyan) and its dimethylamino derivative DMA-NDBF-OH (**2**, magenta) in DMSO. The maximum is shifted bathochromically for photocage **2** due to addition of the N(Me)_2_ moiety and the absorption at 420 nm is 79 times higher.

For 1PE characterisation of DMA-NDBF-OH (**2**) an absorption spectrum was recorded (Fig. 2) in DMSO and compared to the one of unsubstituted NDBF-OH (**1**). The absorption maximum of **2** is shifted bathochromically from 312 to 424 nm. With 15 947 L mol^–1^ cm^–1^ at 424 nm, the molar absorption is 98 times higher than the one of compound **1** (at 420 nm it is 79 times higher).

**Fig. 3:**
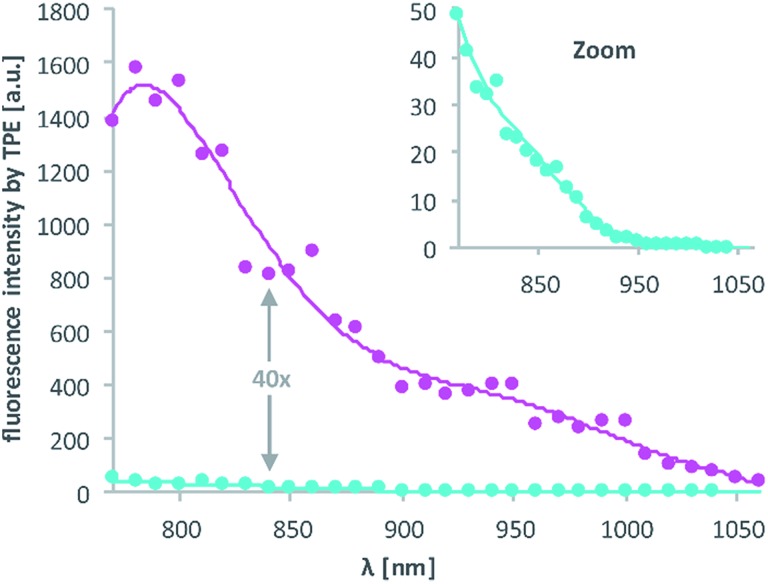
Excitation spectra resulting from 2PE between 770 and 1060 nm of **1** and **2** in DMSO. The excitation power was set to 1.0 mW and squared dependence tested (ESI†). Axes labelling for the “zoom” is the same. Solid lines are averages of the raw data (dots).

Then, 2P-fluorescence excitation (TPE) spectra of **1** and **2** were determined. Fig. 3 shows the relative fluorescence intensities observed in the visible spectral range (∼400–700 nm) after 2PE of **1** (cyan) and **2** (magenta) in DMSO using a wavelength range between 770 and 1060 nm. We observed a generally higher responsiveness to 2PE for **2** than for **1**. The resulting fluorescence intensity for **2** at 840 nm *e.g.* is 40 times higher and should be proportional to the absorption cross section *δ*_a_ of compound **2**. Of course, these 2P-fluorescence excitation spectra do not directly reflect the 2P-uncaging efficiency *δ*_u_ as there is generally no direct relationship between a molecules fluorescence quantum yield *Φ*_f_ and the quantum yield for the uncaging photochemistry *Φ*_u_. However, the observation of high fluorescence intensities after two-photon excitation is a strong indication that compound **2** can indeed be two-photon excited quite readily, which is obviously a decisive prerequisite for any subsequent uncaging photochemistry (for more details see the discussion below and the ESI†).

### Caged oligonucleotides synthesis and characterisation

**Scheme 2:**
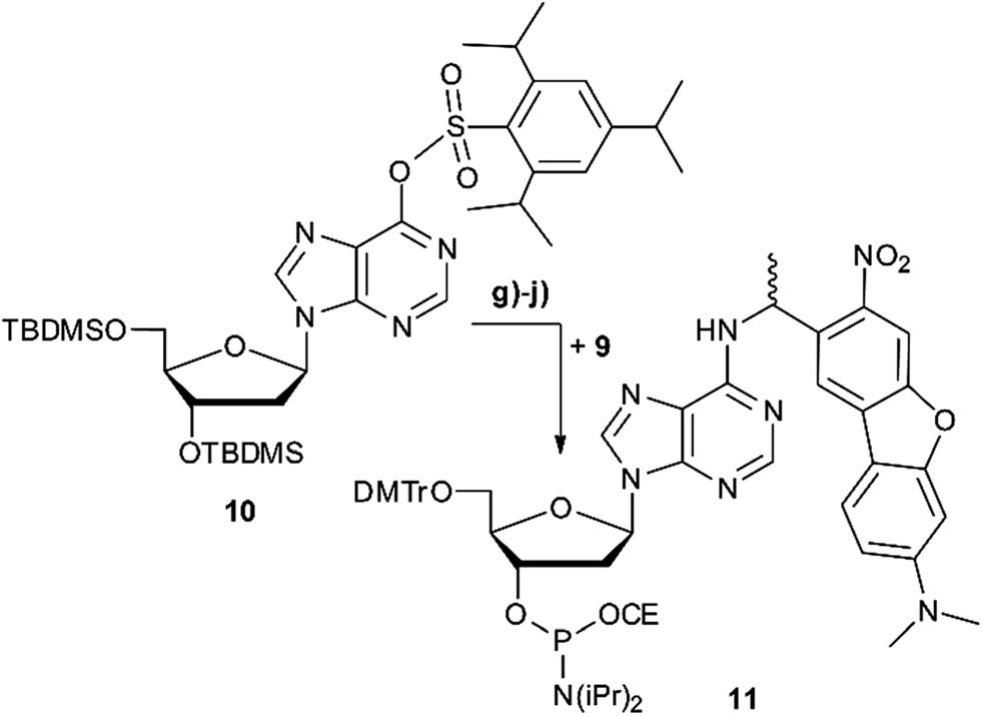
Synthesis scheme of phosphoramidite **11** with new caging group **9**. (g) 4-DMAP, DIPEA, DMF, 2 d, 90 °C, 62%. (h) TBAF, THF, 2 h, rt, 98%. (i) DMTrCl, pyridine, 12 h, rt, 51%. (j) DIPEA, 2-cyanoethyl-*N*,*N*′-diisopropylchlorophosphoramidite, CH_2_Cl_2_, 2 h, rt, 55%. DMAP = (dimethylamino)pyridine, DIPEA = *N*,*N*-diisopropylethylamine, TBAF = tetrabutylammonium fluoride.

Scheme 2 summarises the synthesis of phosphoramidite **11** with the new DMA-NDBF caging group for oligonucleotide solid phase synthesis. Inosine was activated with 2,4,6-triisopropyl-benzenesulfonyl chloride at the nucleobase and 3′ and 5′-OH protected with *tert*-butyldimethylsilyl (TBDMS) before it was reacted with **9**, 4-DMAP and DIPEA in DMF. After a nearly quantitative TBAF-deprotection of the alcohols in THF the 5′-OH was protected with 4,4′-dimethoxytrityl (DMTr). Phosphoramidite **11** was obtained in a reaction with DIPEA and 2-cyanoethyl-*N*,*N*′-diisopropylchlorophosphoramidite in CH_2_Cl_2_.

Photocages NDBF-NH_2_ and DMA-NDBF-NH_2_ (**9**) were introduced in three different sequences – resulting in five different DNA strands (see Table 1).

**Table 1:** The five synthesised DNA strands used in this investigation

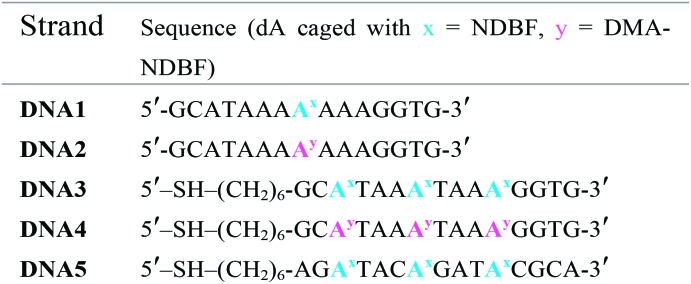

**Fig. 4:**
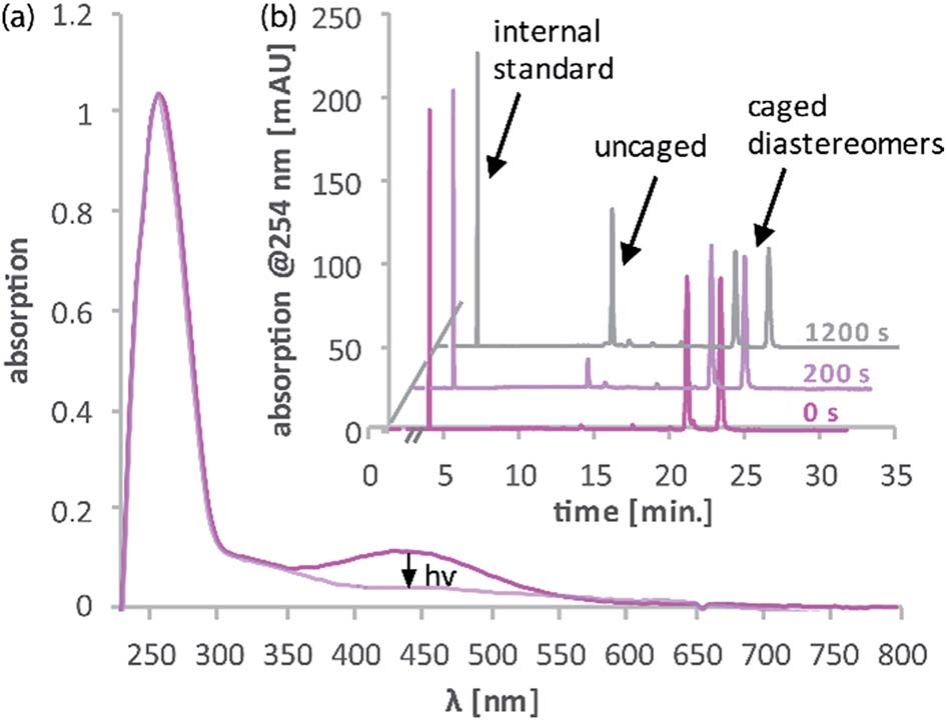
(a) Decrease of absorption between approximately 350 and 540 nm due to irradiation of **DNA2** with dA^DMA-NDBF^ at position 8 and resulting uncaging (0.9 nmol, 45 μl, 365 nm, 0.6 mW). (b) HPLC analysis shows the reaction of the caged diastereomers (*t*_R_ = 21 min. and 23 min.) and increase of the uncaged species (*t*_R_ = 13 min.) (260 pmol, 13 μl, 365 nm, 0.42 mW). With *t*_R_ = retention time on chromatography column.

**Fig. 5:**
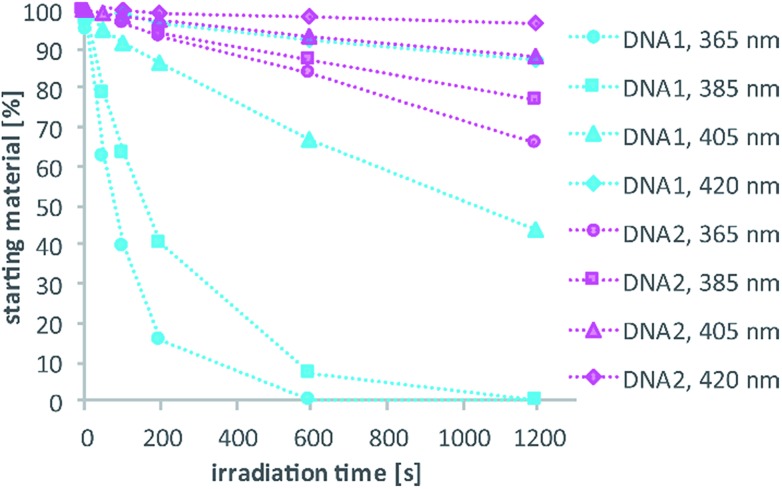
Comparison of 1P-photolysis of **DNA1** and **DNA2** at various wavelengths but with the same number of photons per time. Conversion was determined by HPLC analysis. (260 pmol, 13 μl, 0.42 mW in case of 365 nm irradiation).

Test-sequences **DNA1** and **DNA2**, 15-mers with the cage at position 8, were used for first investigations of 1P-absorption behaviour after irradiation (Fig. 4) and 1P-photolysis (Fig. 5). All irradiation-tests were performed with a concentration of 20 μM in PBS.

Fig. 4a illustrates the absorption decrease of **DNA2** with a dA^DMA-NDBF^ residue between 350 and 540 nm after irradiation with 365 nm. The absorption of the nucleobases at around 260 nm remained unaffected. Quantification of caged and uncaged species could be performed subsequently *via* high-performance liquid chromatography (HPLC; Fig. 4b). With an internal standard (uracil) the amount of starting material could be determined – here demonstrated after irradiation (365 nm, 0.60 mW) for 0, 200 and 1200 seconds. The 1P-photolysis behaviour was tested for **DNA1** and **DNA2** at various wavelengths as shown in Fig. 5. The samples were irradiated for up to 1200 seconds at 365 (0.42 mW), 385 (0.40 mW), 405 (0.38 mW) or 420 nm (0.37 mW). For comparison, the number of photons per time was kept constant.

The 1P-photolysis of **DNA1** was found to be significantly faster at every tested wavelength compared to **DNA2**. For example, after 1200 s irradiation at 385 nm **DNA1** was quantitatively uncaged, whereas 77% starting material remained in case of **DNA2**. After the same time of irradiation at 420 nm we observed 87% remaining starting material for **DNA1** and 97% of **DNA2**, even though DMA-NDBF has its absorption maximum at 424 nm. Further experiments revealed that with wavelengths >420 nm (*i.e.* 455 nm) no 1P-photolysis could be detected at all for **DNA2** – regardless of the power we used! The 1P-quantum yield *Φ*′_420_ for **DNA1** with a dA^NDBF^ residue was found to be 13.6% and for **DNA2** with dA^DMA-NDBF^ 0.05%.^27^ The respective quantum yields *Φ*′_340_ were 24.05% and 1.10%, showing that irradiation into the transition of DMA-NDBF at around 340 nm results in some extent of uncaging whereas the lower-energy transition does not (see also ESI† for a more detailed analysis).

Compound **2** shows some similarity to an amino-substituted *ortho*-nitrobenzyl caging group which has been investigated by Bochet *et al.*^28^ In their case only prior protonation of the amino group led to a state with productive photolysis while irradiation into the unprotonated state yielded a charge transfer transition instead of uncaging. Investigations addressing the charge transfer character in the photochemistry of compound **2** can be found in the ESI.† However, even at pH 2 there was no conversion of **DNA2** upon irradiation at 455 nm.^29^

**Scheme 3:**
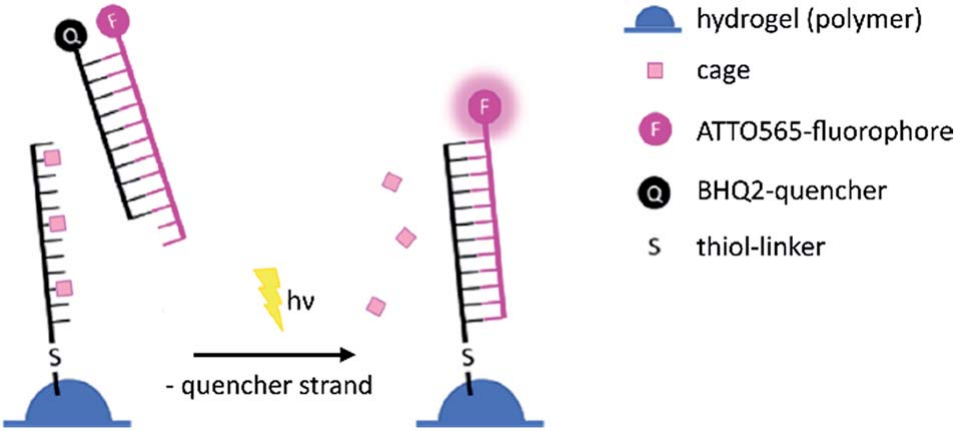
Hydrogel-fluorescence-assay to monitor uncaging. After irradiation with a defined wavelength the triply caged antisense strand becomes uncaged and is able to bind the 5′-fluorophore-labelled strand. By competition, the counter strand with the quencher is removed, the fluorescence increases.

**Fig. 6:**
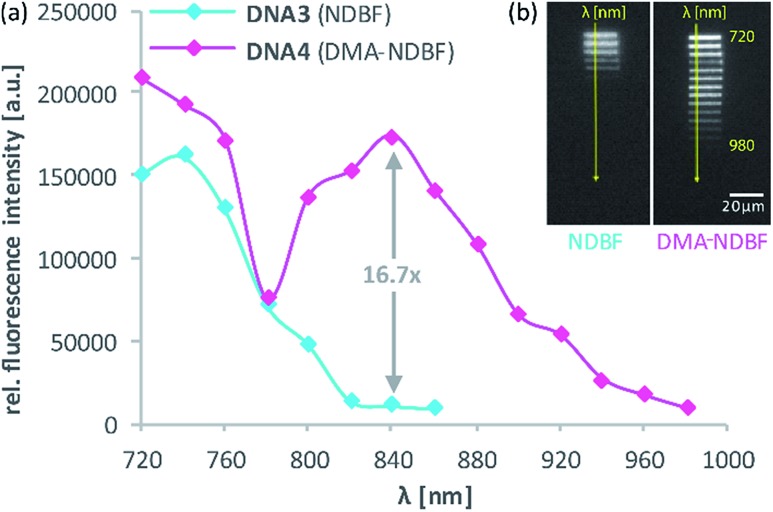
(a) 2P-activation of fluorescence (ATTO565) with NDBF and DMA-NDBF, based on (b) intensity measurements in a hydrogel after uncaging at the indicated wavelengths. DMA-NDBF has a red-shifted local maximum at 840 nm.

For the 2-photon-characterisation of the photocages in DNA strands we used our recently published hydrogel-fluorescence-assay on a confocal microscope and laser setup (ESI†).^30^,^31^ **DNA3** and **DNA4** with either dA^NDBF^ or dA^DMA-NDBF^ residues, respectively, were immobilised *via* thiol-linkers in a hydrogel (PVA-PEG), as illustrated in Scheme 3. Then the gel was soaked with a buffered solution containing a duplex of a 15-mer-strand with a 5′-terminally attached fluorophore (ATTO565) and a 3′-quencher (BHQ2) 11-*mer* strand. The quencher strand displacement after uncaging of **DNA3** or **DNA4** led to increased fluorescence in the focal plane. The uncaging-wavelengths between 720 and 980 nm were generated with a pulsed Ti:sapphire laser for 2P-excitation. For each of the wavelengths investigated one line was written into the hydrogel (Fig. 6b) and the resulting fluorescence was quantified (Fig. 6a). The power was kept constant for every line. The form of the pink spectrum in Fig. 6a (with a maximum at 840 nm) resembles very much the one of the pink spectrum in Fig. 2 (with a maximum at 424 nm). However, 1P-irradiation at 420 nm results in a poor conversion whereas 2P-irradiation at 2 × 420 nm efficiently produces the desired uncaged product strand.

Apparently, DMA-NDBF is one of the few cases where 1PE and 2PE with twice the wavelength do not result in the same photochemical behaviour. In our case, the excited state after 1PE appears to have a low uncaging quantum yield, *i.e.* this caging group can be considered as “one-photon-stable” (at least in the visible range) whereas after 2PE the intended uncaging reaction readily occurs with irradiation conditions that have been shown to be compatible with living cells.^30^

**Fig. 7:**
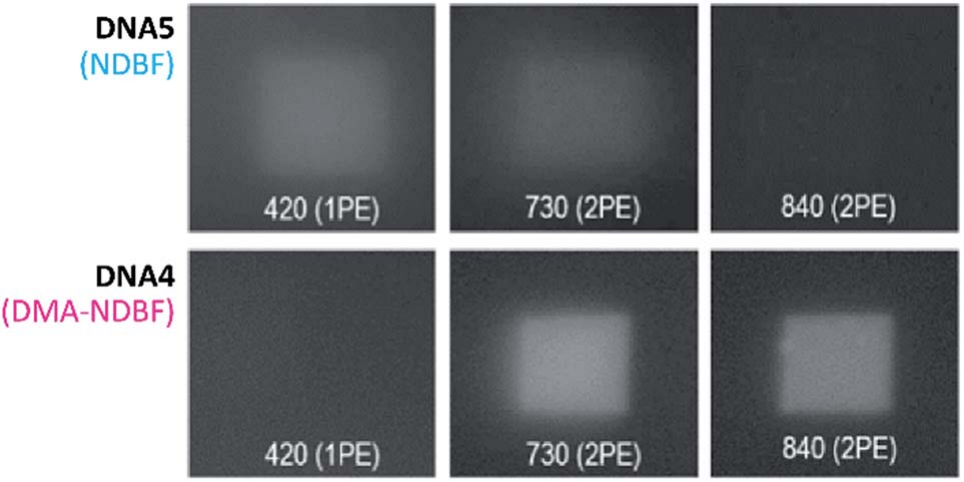
Tests with the triply caged **DNA4** and **DNA5** for optimised uncaging-orthogonality. The best results were obtained with 420 nm with 780 nW and 2 scan repeats, 730 nm and 840 nm with 15 mW and 5 scans.

**Fig. 8:**
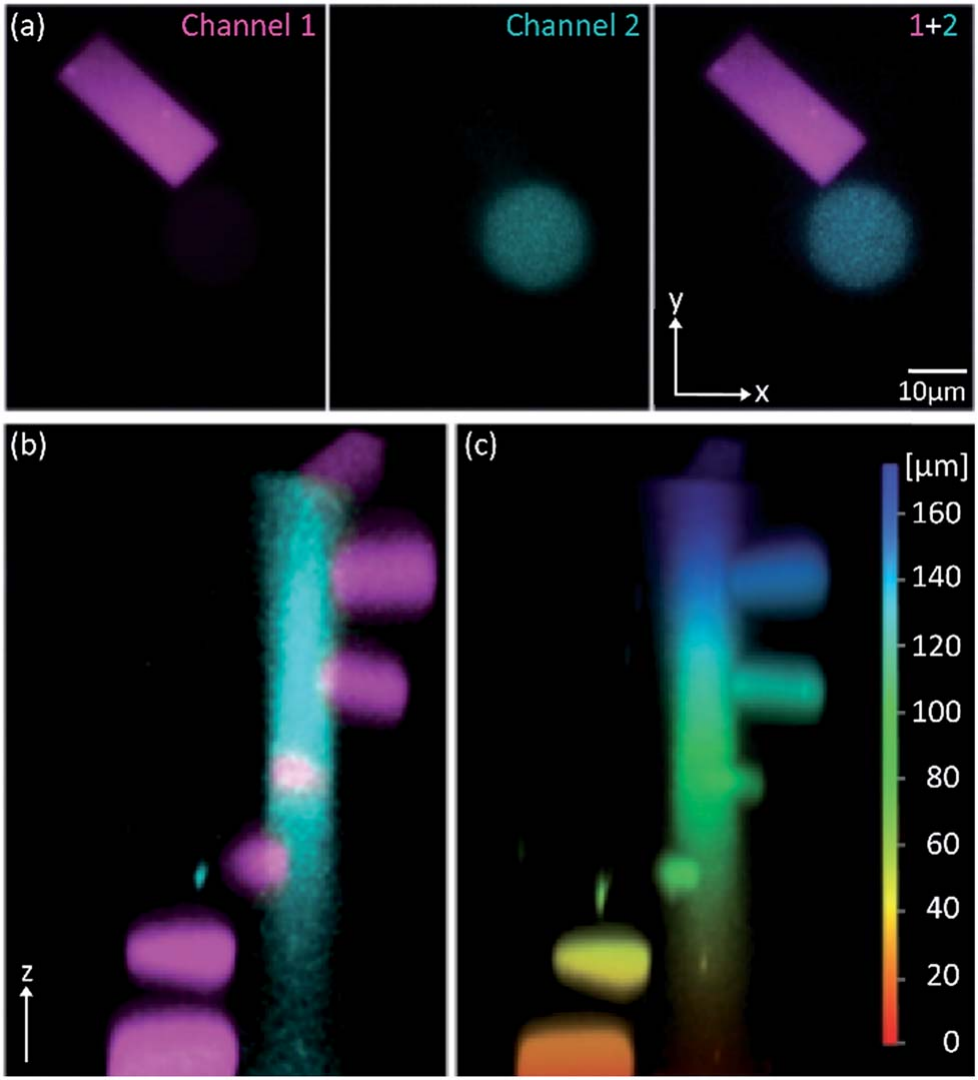
3D picture of a winding staircase with a one-photon uncaging (420 nm) core and two-photon (840 nm) steps. (a) Channel 1, channel 2 and combination in *x*-/*y*-plane and (b) *z*-/*y*-plane. (c) Colour-coded height profile of the *z*-/*y*-plane (0–160 μm).

Because of its low 1P-photolysis rates, we decided to use DMA-NDBF as a “2P-only-cage” which should be applicable for complex orthogonal uncaging together with various 1P-cages – especially also red-shifted ones that are currently the focus of much attention. We used **DNA4** with dA^DMA-NDBF^ residues and **DNA5** with a different sequence and dA^NDBF^ residues for individual addressing together in the same hydrogel. For a two-colour read-out ATTO565 and ATTORho14 were used as fluorophores F1 and F2 and BHQ2 (Q1) and BBQ-650III (Q2) as matching quencher pairs (for an overview see Scheme S2 in the ESI†). Fig. 7 provides an overview of optimised irradiation-conditions, tested for the triply caged strands. With 420 nm (1PE, 780 nW, 2 scans) it was possible to only uncage the NDBF, with 730 nm (2PE, 15 mW, 5 scans) both – however DMA-NDBF much more efficiently than NDBF – and 840 nm (2PE, 15 mW, 5 scans) only DMA-NDBF, while leaving the other cage intact in the same hydrogel. Based on these results, seven rectangular shapes (steps) were written into the hydrogel with 840 nm (Fig. 8), as well as a circular shape with 420 nm. The laser beam direction followed the *z*-axis. The 2P-steps with spatial resolution in *z* had a distance of 25 μm between them. The circular 1P-irradiation resulted in the cylindrical staircase core shown in Fig. 8. A z-stack (Fig. S4†) with two detection channels (Ch. 1: fluorescence excitation 543 nm, detection at 557–612 nm; Ch. 2: excitation 633 nm, detection 671–721 nm) was imaged at laser setup 2 (ESI†). Fig. 8a shows the magenta-channel 1, cyan-channel 2 and the overlaid combination in the *x*/*y*-plane. For Fig. 8b and c the staircase was rotated in the *z*-/*y*-plane. The colours in Fig. 8c demonstrate the height in the hydrogel. The laser powers used were in a range compatible with living cells. For instance, 18–24 mW were found to be tolerated by HEK cells, dorsal root ganglia and liver cells for 10–20 scans.^32^

## Conclusion

In summary we designed, synthesised, and characterised a new dimethylamino derivative of the NDBF photolabile protecting group (PPG). It shows a red-shifted one- and two-photon absorption compared to the NDBF group – which is important for biological applications. As predicted by theoretical calculations the DMA-NDBF PPG shows a better two-photon photolysis behaviour compared to NDBF. However, to our surprise it turned out that the one-photon photolysis efficiency of DMA-NDBF is rather poor, especially for wavelengths beyond 400 nm. Based on our calculations we propose that this is a rare case of excitation-specific photochemistry. Both 1PE and 2PE at twice the wavelength populate the same excited state, since the S_1_ exhibits substantial one-photon oscillator strength as well as two-photon absorption cross section.^26^ However, different photochemical behaviour is induced, because 1PE and 2PE electronic excitations couple to different molecular vibrations.^33^ This unusual “two-photon-only” behaviour offers interesting applications for light-regulation scenarios with increased complexity. We had recently presented orthogonal two-colour, two-photon uncaging^30^ where we had to carefully control the two-photon irradiation conditions (especially the power). With DMA-NDBF, we can now perform efficient two-photon uncaging with red light leaving a broad spectral window open for orthogonal 1P-uncaging in the red part of the spectrum. In addition, 1PE can now be performed before 2PE with DMA-DNBF, which is surprisingly “one-photon-stable”. With previous two-photon caging groups, the 2PE had to be performed as first photochemical operation. This adds another degree of freedom to ever more complex scenarios of complex light control.^34^,^35^ Its one-photon-stability and yet easy and selective photolysis under 2PE, its red-shifted 2P absorption which lies perfectly in the therapeutic window and its perfect stability to regular ambient light make the DMA-NDBF a very interesting caging group for future biological applications.

## Conflicts of interest

There are no conflicts of interest to declare.

## Supplementary Material

Supplementary information
