# Supplementary material for: A red-shifted two-photon-only caging group for three-dimensional photorelease
Source: Chem Sci. 2018 Feb 9;9(10):2797–802. doi: 10.1039/c7sc05182d (PMC5914290; doi:10.1039/c7sc05182d)
Supplement: Supplementary file 1 [file SC-009-C7SC05182D-s001.pdf]

Electronic Supplementary Material (ESI) for Chemical Science.
This journal is © The Royal Society of Chemistry 2018

Electronic Supplementary Information (ESI)

A red-shifted *two-photon-only* caging group for three-dimensional photorelease

Yvonne Becker,‡[a] Erik Unger,‡[a] Manuela A. H. Fichte,[a] Daniel A. Gacek,[b]
Andreas Dreuw,[c] Josef Wachtveitl,[d] Peter J. Walla[b] and
Alexander Heckel*[a]

Table of Contents

1. Small Molecule Synthesis
2. 1P-Absorption Additional Data
3. Emission spectra
4. Wavelength-dependence of the quantum yield for the uncaging of DNA1 and DNA2
5. 2P-Excitation Experiments with NDBF-OH (1) and DMA-NDBF-OH (2)
6. DNA-Synthesis
7. Mass-Spectrometric Characterisation
8. Hydrogel Preparation
9. General Orthogonal Uncaging Scheme
10. Laser Setup 1 "Uncaging Setup"
11. Laser Setup 2 "Imaging Setup"
12. References
13. ^{1}H-NMR spectra
14. Mass spectra

1. Small Molecule Synthesis

General synthesis methods All reactions were performed under a protective argon atmosphere unless otherwise specified. Reagents and solvents were purchased from commercial sources and used without further purification. Deionised (DI) water was used for all experiments. Reaction progresses were monitored with silica gel 60-coated TLC-sheets and reaction product purifications via flash chromatography were performed with silica gel 60, both by *Macherey-Nagel*. ^{1}H, ^{13}C and ^{31}P-NMR spectra were recorded on a *Bruker* AV400 or AV500 MHz spectrometer. Mass spectra (MS) were obtained using a "Surveyor MSQ" for LC-ESI measurements and high resolution mass spectra (HRMS) were obtained with a *ThermoScientific* "LTQ Orbitrap XL" (MALDI-HRMS). For elemental analysis a "vario MICRO cube" from *Elementar* was used.

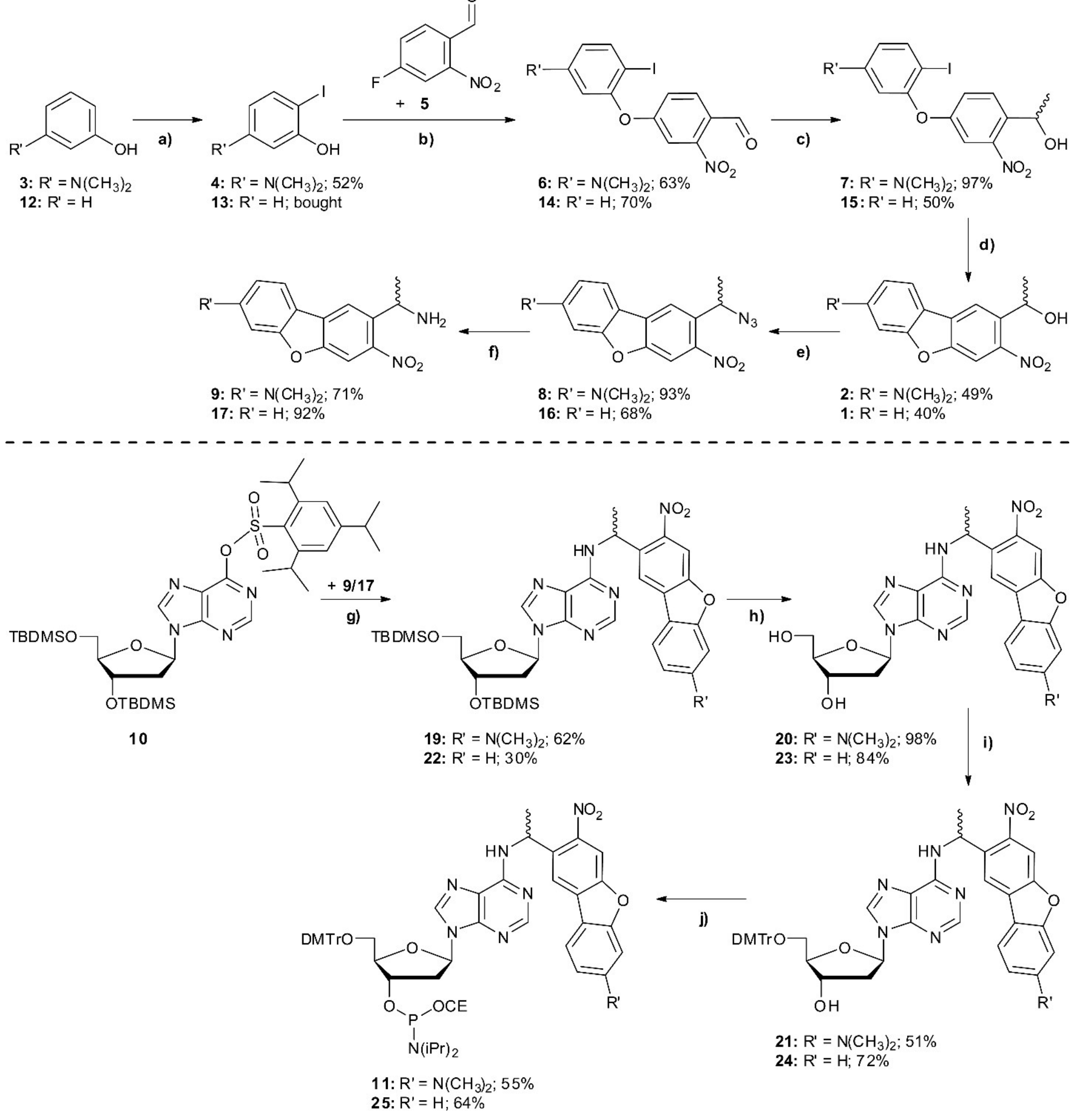

Scheme S1: Small molecule synthesis of phosphoramidite **11** and **25** (bottom) with DMA-NDBF-NH_2 **9** and NDBF-NH_2 **17** (top).

The activated deoxyadenosine (compound **10**) was synthesised in analogy to Schäfer *et al.* "Wavelength-selective uncaging of dA and dC residues".[1] The NDBF-OH (compound **1**) synthesis was based on a previous publication by Deiters *et al.* "Improved synthesis of the two-photon caging group 3-nitro-2-ethyldibenzofuran and its application to a caged thymidine phosphoramidite".[2]

NDBF (R' = H)

2-(1-Azidoethyl)-3-nitrodibenzo[*b,d*]furan (16): A cold solution of 0.56 g (8.57 mmol, 4.0 eq.) sodium azide in 2 ml DI H_2O was covered with 2 ml toluene in a test tube and acidified with conc. H_2SO_4 to generate HN_3 *in situ*. The organic layer was extracted with a syringe, dried over $MgSO_4$ and added dropwise to 0.55 g (2.14 mmol, 1.0 eq.) 1-(3-nitrodibenzo[*b,d*]furan-2-yl)ethanol and 0.84 g (3.21 mmol, 1.5 eq.) PPh_3 which had been dissolved in 20 ml dry THF and cooled down to 0 °C. After 10 min stirring, 1.5 ml (3.21 mmol, 1.5 eq.) DEAD were added and the solution was allowed to heat up to room temperature and stirred for further 12 h. The crude product was dried under reduced pressure, purified via column chromatography (cyclohexane:EtOAc = 40:1) and recrystallised (cyclohexane) to obtain azide **16** as a colourless solid (0.40 g, 68%). R_f (cyclohexane:EtOAc = 20:1) 0.54. ^{1}H-NMR (500 MHz, DMSO-d$_6$) δ [ppm] = 8.61 (s, 1H), 8.43 (s, 1H), 8.39 (d, 1H, *J* = 8 Hz), 7.82 (d, 1H, *J* = 1 Hz), 7.68 (t, 1H, *J* = 8 Hz), 7.52 (t, 1H, *J* = 8 Hz), 5.46 (q, 1H, *J* = 7 Hz), 1.66 (d, 3H, *J* = 7 Hz). ^{13}C-NMR (125.8 MHz, DMSO-d$_6$) δ [ppm] = 157.6, 153.4, 147.0, 130.7, 129.9, 128.2, 124.1, 122.8, 122.0, 120.9, 112.2, 108.6, 55.8, 21.6. Elemental anal. found: C, 59.00; H, 3.77; N, 20.04. $C_{14}H_{10}N_4O_3$ requires C, 59.57; H, 3.57; N, 19.85%.

2-(1-Aminoethyl)-3-nitrodibenzo[*b,d*]furan (17): 1.09 g (3.86 mmol, 1.0 eq.) azide **16** and 1.01 g (3.86 mmol, 1.0 eq.) PPh_3 were dissolved in 15 ml of a 1:1 mixture of THF and MeCN, stirred at room temperature until the solution cleared up and then stirred for 3.5 h at 70 °C. 20 ml DI H_2O were added before the solution was stirred at room temperature overnight. Again 5 ml DI H_2O were added and stirring continued for 3 h. The solvents were removed under reduced pressure and the residue dissolved in EtOAc. The organic layer was washed with DI H_2O and brine. The aqueous layer was extracted twice with EtOAc and the combined organic layers dried over $MgSO_4$.The crude product was dried under reduced pressure, purified via column chromatography (CH_2Cl_2:MeOH = 100:1 + 0.1% NEt_3) and recrystallised (cyclohexane) to obtain **17** as a bright yellow solid (0.90 g, 92%). R_f (CH_2Cl_2:MeOH = 9:1) 0.37. ^{1}H-NMR (500 MHz, DMSO-d$_6$) δ [ppm] = 8.67 (s, 1H), 8.27 (d, 1H, *J* = 8 Hz), 8.25 (s, 1H), 7.79 (d, 1H, *J* = 8 Hz), 7.66-7.63 (m, 1H), 7.51-7.48 (m, 1H), 4.45 (q, 1H, *J* = 7 Hz), 2.10 (s, 2H), 1.41 (d, 3H, *J* = 7 Hz). ^{13}C-NMR (125.8 MHz, DMSO-d$_6$) δ [ppm] = 157.4, 152.6, 147.4, 138.1, 129.4, 127.6, 123.9, 122.3, 122.2, 120.2, 112.1, 107.3, 46.1, 25.8. MALDI-HRMS *m/z* calcd. for $C_{14}H_{13}N_2O_3$ $[M+H]^+$ 257.09207, found 257.09163 (Δm = 0.00044, error 1.7ppm).

3',5'-*O*-Di(*tert*buthyldimethylsilyl)-6-*N*-[1-(3-nitrodibenzo[*b,d*]furan-2-yl)ethyl]-2'-deoxyadenosin (22) 2.72 g (3.64 mmol, 1.03 eq.) *O*^{6}-(2,4,6-triisopropylbenzenesulfonyl)-3',5'-*O*-di(*tert*butyldimethylsilyl)-2'-deoxyinosin **10**, 0.91 g (3.52 mmol, 1.00 eq.) amine **17**, 0.43 g (3.52 mmol, 1.00 eq.) 4-DMAP and 1.6 ml (9.16 mmol, 2.60 eq.) DIPEA were dissolved in 12 ml dry DMF and stirred for 2 h at room temperature and 3 d at 90 °C. The crude product was dried under reduced pressure and the residue dissolved in CH_2Cl_2. The organic layer was washed once with 5% citric acid and twice with saturated solution of $NaHCO_3$ and the aqueous layer was extracted with CH_2Cl_2. Combined organic layers were dried over $MgSO_4$, solvent removed under reduced pressure and purification was performed via column chromatography (cyclohexane:EtOAc = 5:1 ⟶ 4:1 ⟶ 2:1). **22** was obtained as yellow foam (745 mg, 30%). R_f (cyclohexane:EtOAc = 3:1) 0.29. ^{1}H-NMR (500 MHz, DMSO-d$_6$) δ [ppm] = 8.59 (m, 2H), 8.32-8.12 (m, 2H), 8.04-8.01 (m, 2H), 7.71 (d, 1H, *J* = 8 Hz), 7.58 (t, 1H, *J* = 8 Hz), 7.43 (t, 1H, *J* = 8 Hz), 6.36-5.87 (m, 2H), 4.62-4.52 (m, 1H), 3.76-3.70 (m, 2H), 3.59-3.56 (m, 1H), 2.91-2.84 (m, 1H), 2.22 (m, 1H), 1.75 (d, 3H, *J* = 7 Hz), 0.83-0.62 (m, 18H), 0.04 (s, 6H), -0.06-(-0.18) (m, 6H). ^{13}C-NMR (125.8 MHz, DMSO-d$_6$) δ [ppm] = 157.4, 152.7, 152.7, 152.0, 148.6, 148.4, 147.9, 147.8, 139.9, 139.5, 129.5, 127.8, 123.9, 121.9, 121.7, 120.0, 119.7, 112.1, 107.5, 107.3, 86.9, 86.7, 83.3, 71.8, 71.6, 62.4, 62.1, 44.8, 38.5, 32.5, 29.0, 25.6, 25.5, 25.4, 22.4, 17.9, 17.6, 17.6, -4.9, -5.1, -5.1, -5.7, -5.8, -5.8. MALDI-HRMS *m/z* calcd. for $C_{36}H_{51}N_6O_6Si_2$ $[M+H]^+$ 719.34031, found 719.34059 (Δm = 0.00028, error 0.4ppm).

6-*N*-[1-(3-Nitrodibenzo[*b,d*]furan-2-yl)ethyl]-2'-deoxyadenosin (23) 0.75 g (1.04 mmol, 1.0 eq.) **22** were dissolved in 20 ml dry THF and 2.1 ml (2.07 mmol, 2.0 eq.) of 1M TBAF/THF solution added dropwise. After full conversion of **22** the solvent was removed under reduced pressure, the residue redissolved in CH_2Cl_2 and washed with saturated $NaHCO_3$-solution. The aqueous layer was extracted twice with CH_2Cl_2 and dried over $MgSO_4$. The crude product was dried under reduced pressure and purified via column chromatography (CH_2Cl_2:MeOH = 40:1 ⟶ 20:1 ⟶ 10:1) to obtain **23** as a yellow solid (415 mg, 84%). R_f (CH_2Cl_2:MeOH = 20:1) 0.38. ^{1}H-NMR (500 MHz, DMSO-d$_6$) δ [ppm] = 8.61 (m, 2H), 8.39-8.07 (m, 4H), 7.75 (d, 1H, *J* = 8 Hz), 7.61 (t, 1H, *J* = 8 Hz), 7.45 (t, 1H, *J* = 8 Hz), 6.39-5.90 (m, 2H), 5.27 (m, 1H), 5.13-5.10 (m, 1H), 4.38-4.37 (m, 1H), 3.84 (m, 1H), 3.59-3.57 (m, 1H), 3.50-3.46 (m, 1H), 2.67-2.63 (m, 1H), 2.21 (m, 1H), 1.76 (d, 3H, *J* = 7 Hz). ^{13}C-NMR (125.8 MHz, DMSO-d$_6$) δ [ppm] = 157.4, 152.8, 152.0, 148.5, 147.8, 139.7, 135.8, 129.6, 127.8, 124.0, 121.9, 121.8, 120.0, 119.7, 112.2, 107.6, 87.9, 87.9, 83.8, 70.9, 70.8, 61.8, 61.8, 44.9, 21.8. MALDI-HRMS *m/z* calcd. for $C_{24}H_{23}N_6O_6$ $[M+H]^+$ 491.16736, found 491.16689 (Δm = 0.00047, error 1.0ppm).

5'-*O*-(4,4'-Dimethoxytrityl)-6-*N*-[1-(3-nitrodibenzo[*b,d*]furan-2-yl)ethyl]-2'-deoxyadenosin (24) 337 mg (0.69 mmol, 1.0 eq.) **23** were coevaporated twice with 10 ml dry pyridine and then dissolved in 20 ml. 303 mg (0.89 mmol, 1.3 eq.) 4,4'-dimethoxytrityl chloride (DMTrCl)

were added in portions and stirred for 3 h at room temperature. Afterwards further 303 mg DMTrCl were added to the solution and stirred overnight. The solvent was removed under reduced pressure, the residue redissolved in CH_2Cl_2 and washed with 5% citric acid and then saturated $NaHCO_3$-solution. The aqueous layers were extracted with CH_2Cl_2, dried over $MgSO_4$ and the solvent removed under reduced pressure. Purification was performed via column chromatography (CH_2Cl_2:MeOH = 50:1 → 20:1) to obtain **24** as a yellow foam (389 mg, 72%). R_f (CH_2Cl_2:MeOH = 20:1) 0.23. ^{1}H-NMR (500 MHz, DMSO-d$_6$) δ [ppm] = 8.61 (m, 2H), 8.32-7.96 (m, 4H), 7.77 (d, 1H, *J* = 8 Hz), 7.62 (t, 1H, *J* = 8 Hz), 7.46 (t, 1H, *J* = 8 Hz), 7.28-7.27 (m, 2H), 7.20-7.04 (m, 7H), 6.79-6.75 (m, 4H), 6.40-5.88 (m, 2H), 5.33-5.30 (m, 1H), 4.46-4.37 (m, 1H), 3.94 (m, 1H), 3.70 (s, 6H), 3.16-3.12 (m, 2H), 2.89-2.79 (m, 1H), 2.27-2.26 (m, 1H), 1.75 (d, 3H, *J* = 7 Hz). ^{13}C-NMR (125.8 MHz, DMSO-d$_6$) δ [ppm] = 158.0, 158.0, 157.5, 152.8, 152.1, 144.8, 135.8, 135.6, 135.5, 135.4, 129.7, 129.6, 129.6, 127.9, 127.6, 127.6, 126.5, 124.0, 121.9, 121.8, 113.1, 112.2, 85.8, 85.4, 83.4, 70.6, 64.0, 55.0, 21.9. MALDI-HRMS *m/z* calcd. for $C_{45}H_{41}N_6O_8$ [M+H]$^+$ 793.29804, found 793.29809 (Δm = 0.00005, error 0.1ppm).

3'-*O*-(2-Cyanoethoxy-*N,N'*-diisopropylamino)phosphine-5'-*O*-(4,4'-dimethoxytrityl)-6-*N*-[1-(3-nitrodibenzo[*b,d*]furan-2-yl)ethyl]-2'-deoxyadenosin (25) 368 mg (0.46 mmol, 1.0 eq.) **24** and 0.4 ml (2.32 mmol, 5.0 eq.) DIPEA were dissolved in 2.5 ml dry CH_2Cl_2 and stirred for 25 min at room temperature before 0.2 ml (0.93 mmol, 2.0 eq.) 2-cyanoethyl-*N,N'*-diisopropylchlorophosphoramidite were added. After 2 h the solution was diluted with CH_2Cl_2 to a total volume of 50 ml, washed with saturated $NaHCO_3$-solution and the aqueous layers were extracted twice with CH_2Cl_2. The combined organic layers were dried over $MgSO_4$ and the solvent removed under reduced pressure. The crude product was purified via column chromatography (cyclohexane:EtOAc = 2:1 → 1:1 → 1:2) and four times precipitation with *n*-hexane from acetone. **25** was obtained as a yellow foam (294 mg, 64%). R_f (cyclohexane:EtOAc = 1:1) 0.36 diastereomer 1, 0.25 diastereomer 2. ^{1}H-NMR (500 MHz, DMSO-d$_6$) δ [ppm] = 8.63-8.59 (m, 2H), 8.32-7.94 (m, 4H), 7.76 (d, 1H, *J* = 8 Hz), 7.61 (t, 1H, *J* = 8 Hz), 7.44 (m, 1H), 7.29-7.25 (m, 2H), 7.16-7.00 (m, 7H), 6.78-6.73 (m, 4H), 6.34-5.87 (m, 2H), 4.73 (m, 1H), 4.09-4.05 (m, 1H), 3.70-3.60 (m, 8H), 3.55-3.47 (m, 2H), 3.22-3.12 (m, 2H), 3.01-3.00 (m, 1H), 2.73 (t, 1H, *J* = 6 Hz), 2.65 (t, 1H, *J* = 6 Hz), 2.41 (m, 1H), 1.75 (d, 3H, *J* = 7 Hz), 1.12-0.99 (m, 12H). ^{13}C-NMR (125.8 MHz, DMSO-d$_6$) δ [ppm] = 158.0, 157.9, 157.9, 157.4, 153.1, 152.7, 152.0, 148.4, 147.7, 144.6, 140.2, 139.9, 135.8, 135.5, 135.3, 135.3, 135.2, 135.1, 129.7, 129.7, 129.6, 129.5, 127.9, 127.6, 127.5, 126.5, 126.4, 124.0, 121.9, 121.8, 120.0, 119.9, 119.8, 119.0, 118.7, 113.0, 112.2, 107.6, 85.5, 85.5, 84.8, 84.5, 83.5, 73.3, 73.2, 72.6, 63.4, 63.2, 58.5, 58.4, 58.4, 58.3, 54.9, 44.9, 42.6, 42.6, 42.5, 42.5, 37.2, 24.3, 24.2, 24.2, 24.2, 24.1, 24.1, 24.0, 21.9, 19.8, 19.7, 19.6. ^{31}P-NMR (202. 5 MHz, DMSO-d$_6$) δ [ppm] = 147.6, 147.6, 147.5, 147.5, 147.0, 147.0, 147.0, 146.9. ESI-MS *m/z* calcd. for $C_{54}H_{58}N_8O_9P$ [M+H]$^+$ 993.41, found 993.49.

DMA-NDBF (R' = $N(CH_3)_2$)

2-Iodo-5-dimethylaminophenol (4) 5.00 g (36.45 mmol, 1.0 eq.) 3-dimethylaminophenol were suspended in 200 ml 0.1 N H_2SO_4. Two solutions were added simultaneously but separately: one contained 4.05 g (24.40 mmol, 0.7 eq.) KI in 100 ml DI H_2O, the other 2.60 g (12.15 mmol, 0.3 eq.) KIO_3 in 1.2 ml conc. H_2SO_4 and 100 ml DI H_2O. After stirring at room temperature for 2 h the pH-value was adjusted to 1.5 with conc. H_2SO_4. The black precipitate was filtered off and the filtrate neutralised with Na_2CO_3. After a second filtration the solid residue was dried under reduced pressure. 2-iodo-5-dimethylaminophenol was obtained as a grey solid (4.94 g, 52%). R_f (cyclohexane:EtOAc = 5:1) 0.31. ^{1}H-NMR (500 MHz, DMSO-d$_6$) δ [ppm] = 9.89 (s, 1H), 7.34 (d, 1H, *J* = 9 Hz), 6.26 (d, 1H, *J* = 3 Hz), 6.06 (dd, 1H, *J* = 9 Hz, *J* = 3 Hz), 2.83 (s, 6H). ^{13}C-NMR (125.8 MHz, DMSO-d$_6$) δ [ppm] = 156.9, 151.7, 138.2, 106.7, 99.4, 67.9, 40.0. MALDI-HRMS *m/z* calcd. for $C_8H_{11}INO$ [M+H]$^+$ 263.98798, found 263.98798 (Δm = 0.00000, error 0.0ppm).

4-(2-Iodo-5-dimethylaminophenoxy)-2-nitrobenzaldehyde (6) 0.20 g (1.80 mmol, 1.05 eq.) KO*t*Bu were added to a solution of 0.45 g (1.71 mmol, 1.0 eq.) 2-iodo-5-dimethyl-aminophenol in dry DMSO and stirred for 1 h at room temperature. Afterwards 0.32 g (1.88 mmol, 1.10 eq.) 4-fluoro-2-nitrobenzaldehyde were dissolved in 10 ml DMSO and added dropwise. The mixture was stirred further 48 h and then concentrated under vacuum. The residue was dissolved in 200 ml ethyl acetate and washed with water and brine. The aqueous layer was extracted with ethyl acetate and the combined organic layers were dried over Na_2SO_4. Via column chromatography (cyclohexane:EtOAc = 20:1 -> 9:1) the crude product was purified and obtained as red solid (0.44 g, 63%). R_f (cyclohexane:EtOAc = 4:1) 0.40. ^{1}H-NMR (500 MHz, DMSO-d$_6$) δ [ppm] = 10.08 (s, 1H), 7.98 (d, 1H, *J* = 9 Hz), 7.66 (d, 1H, *J* = 9 Hz), 7.54 (d, 1H, *J* = 3 Hz), 7.24 (dd, 1H, *J*= 9 Hz, *J* = 3 Hz), 6.64 (d, 1H, *J* = 3 Hz), 6.54 (dd, 1H, *J* = 9 Hz, *J* = 3 Hz), 2.90 (s, 6H). ^{13}C-NMR (125.8 MHz, DMSO-d$_6$) δ [ppm] = 188.3, 161.3, 153.6, 152.2, 150.9, 139.5, 132.8, 123.7, 119.9, 112.7, 111.6, 106.0, 71.5, 39.9. MALDI-HRMS *m/z* calcd. for $C_{15}H_{14}IN_2O_4$ [M+H]$^+$ 412.99928, found 412.99836 (Δm = 0.00092, error 2.2ppm).

1-[4-(2-Iodo-5-dimethylaminophenoxy)-2-nitrophenyl]ethanol (7) 200 mg (0.49 mmol, 1.0 eq.) benzaldehyde **6** were dissolved in 5 ml dry CH_2Cl_2 and cooled down to 0 °C before 0.5 ml (0.97 mmol, 2.0 eq.) $AlMe_3$ were added dropwise. The mixture was stirred 5 min at 0 °C and then allowed to heat up to room temperature. The reaction was quenched by adding 10 ml 1 M NaOH and the organic layer was washed once with water, once with brine. The aqueous layer was extracted with CH_2Cl_2 and the combined organic layers dried over Na_2SO_4. Via column chromatography (cyclohexane:EtOAc = 4:1 -> 2:1) the crude product was purified and **7** obtained as red solid (199 mg, 97%). R_f (cyclohexane:EtOAc = 3:1) 0.31. ^{1}H-NMR (500 MHz, DMSO-d$_6$) δ [ppm] = 7.78 (d, 1H, *J* = 9 Hz), 7.62 (d, 1H, *J* = 9 Hz), 7.26 (d, 1H, *J* = 3 Hz), 7.20 (dd, 1H, *J* = 9 Hz, *J* = 3 Hz), 6.53 (d, 1H, *J* = 3 Hz), 6.50 (dd, 1H, *J* = 9 Hz, J = 3 Hz), 5.47 (d, 1H, *J* = 4 Hz), 5.07-5.03 (m, 1H), 2.88 (s, 6H), 1.36 (d, 3H, *J* = 9 Hz). ^{13}C-NMR (125.8 MHz, DMSO-d$_6$) δ [ppm] = 156.2, 154.3, 152.2, 148.0, 139.3, 135.4, 129.5, 120.9, 112.2, 110.5, 105.8, 72.1, 63.6, 39.9, 25.1. MALDI-HRMS *m/z* calcd. for $C_{16}H_{18}IN_2O_4$ [M+H]$^+$ 429.03058, found 429.02958 (Δm = 0.00100, error 2.3ppm).

1-[7-(Dimethylamino)-3-nitrodibenzo[*b,d*]furan-2-yl]ethanol (2) 1.34 g (4.11 mmol, 2.0 eq.) $CsCO_3$ and 0.09 g (0.41 mmol, 0.2 eq.) $Pd(OAc)_2$ were added to a solution of 0.88 g (2.06 mmol, 1.0 eq.) **7** dissolved in 75 ml degassed DMAc. Afterwards 1.0 ml degassed H_2O were added and the mixture stirred for 72 h at 80 °C. Then the mixture was filtered over celite, washed with EtOAc and dried under reduced pressure. The solid residue was recrystallised to obtain the closed ring-form **2** as a red solid (300 mg, 49%). R_f (cyclohexane:EtOAc = 3:1) 0.31. ^{1}H-NMR (500 MHz, DMSO-d_6) δ [ppm] = 8.31 (s, 1H), 8.17 (s, 1H), 8.04 (d, 1H, *J* = 9 Hz), 6.94 (d, 1H, *J* = 2 Hz), 6.89 (dd, 1H, *J* = 9 Hz, *J* = 2 Hz), 5.55 (d, 1H, *J* = 4 Hz), 5.33-5.29 (m, 1H), 3.06 (s, 6H), 1.46 (d, 3H, *J* = 6 Hz). ^{13}C-NMR (125.8 MHz, DMSO-d_6) δ [ppm] = 160.5, 152.4, 152.2, 143.5, 138.5, 129.6, 122.6, 117.2, 110.4, 109.8, 106.9, 93.6, 64.2, 40.3, 25.5. MALDI-HRMS *m/z* calcd. for $C_{16}H_{17}N_2O_4$ $[M+H]^+$ 301.11828, found 301.11828 (Δm = 0.00000, error 0.0ppm).

2-(1-Azidoethyl)-7-(dimethylamino)-3-nitrodibenzo[*b,d*]furan (8): An ice-cold solution of 86 mg (1.33 mmol, 4.0 eq.) sodium azide in 1 ml DI H_2O was covered with 1 ml toluene in a test tube and acidified with conc. H_2SO_4 to generate HN_3 *in situ*. The organic layer was extracted with a syringe, dried over $MgSO_4$ and added dropwise to 100 mg (0.33 mmol, 1.0 eq.) **2** and 131 mg (0.50 mmol, 1.5 eq.) PPh_3, which had been dissolved in 5 ml dry THF and cooled down to 0 °C. After 10 min stirring 0.2 ml (0.50 mmol, 1.5 eq.) DEAD were added and the solution was allowed to heat up to room temperature and stirred for further 12 h. The crude product was dried under reduced pressure and purified via column chromatography (cyclohexane:EtOAc = 4:1). Azide **8** was obtained as a red solid (100 mg, 93%). R_f (cyclohexane:EtOAc = 3:1) 0.56. ^{1}H-NMR (500 MHz, DMSO-d_6) δ [ppm] = 8.29 (s, 1H), 8.26 (s, 1H), 8.08 (d, 1H, *J* = 9 Hz), 6.95 (d, 1H, *J* = 2 Hz), 6.92 (dd, 1H, *J* = 9 Hz, *J* = 2 Hz), 5.51 (q, 1H), 3.07 (s, 6H), 1.63 (d, 3H, *J* = 7 Hz). ^{13}C-NMR (125.8 MHz, DMSO-d_6) δ [ppm] = 160.6, 152.8, 152.4, 144.2, 131.4, 129.9, 122.9, 118.1, 110.1, 110.0, 107.7, 93.4, 56.0, 40.2, 21.6. MALDI-HRMS *m/z* calcd. for $C_{16}H_{16}N_5O_3$ $[M+H]^+$ 326.12477, found 326.12409 (Δm = 0.00068, error 2.1ppm). Elemental anal. found: C, 59.12; H, 4.77; N, 21.74. $C_{16}H_{15}N_5O_3$ requires C, 59.07; H, 4.65; N, 21.53%.

2-[(1-Aminoethyl)-7-(dimethylamino)]-3-nitrodibenzo[*b,d*]furan (9): 1.05 g (3.23 mmol, 1.0 eq.) azide **8** and 0.85 g (3.23 mmol, 1.0 eq.) PPh_3 were dissolved in 14 ml of a 1:1 mixture of THF and MeCN, stirred for 40 min at room temperature, for 3 h at 70 °C and after addition of 7 ml DI H_2O for further 3 h at 70 °C and 12 h at room temperature. The solvents were removed under reduced pressure and the residue dissolved in EtOAc. The organic layer was washed twice with DI H_2O and once with brine. The aqueous layer was extracted twice with EtOAc and the combined organic layers dried over Na_2SO_4.The crude product was dried under reduced pressure and purified via column chromatography (CH_2Cl_2:MeOH = 40:1 +0.1% NEt_3) to obtain amine **9** as a red solid (0.68 g, 71%). R_f = (CH_2Cl_2:MeOH = 9:1) 0.48. ^{1}H-NMR (500 MHz, DMSO-d_6) δ [ppm] = 8.39 (s, 1H), 8.09 (s, 1H), 7.97 (d, 1H, *J* = 9 Hz), 6.94 (d, 1H, *J* = 2 Hz), 6.90 (dd, 1H, *J* = 9 Hz, *J* = 2 Hz), 4.50 (q, 1H, *J* = 6 Hz), 3.06 (s, 6H), 2.09 (br s, 2H), 1.39 (d, 3H, *J* = 7 Hz). ^{13}C-NMR (125.8 MHz, DMSO-d_6) δ [ppm] = 160.3, 152.1, 152.1, 144.6, 139.1, 129.2, 122.3, 117.6, 110.5, 109.7, 106.7, 93.7, 46.1, 40.3, 25.7. MALDI-HRMS *m/z* calcd. for $C_{16}H_{18}N_3O_3$ $[M+H]^+$ 300.13427, found 300.13435 (Δm = 0.00008, error 0.3ppm).

3′,5′-*O*-Di(*tert*buthyldimethylsilyl)-6-*N*-{1-[(7-dimethylamino)-3-nitrodibenzo[*b,d*]furan-2-yl]ethyl}-2′-deoxyadenosin (19):
2.10 g (2.81 mmol, 1.50 eq.) O^6-(2,4,6-triisopropylbenzenesulfonyl)-3′,5′-di-*O*-(tertbutyldimethylsilyl)-2′-deoxyinosin, 0.56 g (1.87 mmol, 1.00 eq.) amine **9**, 0.23 g (1.87 mmol, 1.00 eq.) 4-DMAP and 0.8 ml (4.86 mmol, 2.60 eq.) DIPEA were dissolved in 18 ml dry DMF and stirred for 48 h at 90 °C. The crude product was dried under reduced pressure and the residue dissolved in CH_2Cl_2. The organic layer was washed twice with 5% citric acid and saturated solution of $NaHCO_3$ and the aqueous layer was extracted with CH_2Cl_2. Combined organic layers were dried over Na_2SO_4, solvent removed under reduced pressure and purification was performed via column chromatography (cyclohexane:EtOAc = 4:1). **19** was obtained as red foam (0.89 g, 62%). R_f = (cyclohexane:EtOAc = 3:1) 0.24. ^{1}H-NMR (500 MHz, DMSO-d_6) δ [ppm] = 8.56 (m, 1H), 8.34-8.02 (m, 4H), 7.78 (d, 1H, *J* = 9 Hz), 6.90 (s, 1H), 6.85 (dd, 1H, *J* = 9 Hz, *J* = 2 Hz), 6.39-5.93 (m, 2H), 4.63-4.58 (m, 1H), 3.79-3.73 (m, 2H), 3.62-3.57 (m, 1H), 3.03 (s, 6H), 2.94-2.84 (m, 1H), 2.25 (m, 1H), 1.72 (d, 3H, *J* = 3 Hz), 0.87-0.69 (m, 18H), 0.08 (s, 6H), -0.02 – (-0.12) (m, 6H). ^{13}C-NMR (125.8 MHz, DMSO-d_6) δ [ppm] = 160.2, 153.2, 152.2, 152.0, 148.5, 148.4, 145.0, 139.7, 139.4, 129.3, 121.8, 117.2, 110.0, 109.6, 106.7, 106.6, 93.5, 86.8, 86.7, 83.3, 83.2, 71.8, 71.6, 62.3, 62.1, 44.9, 40.1, 38.4, 26.2, 25.6, 25.4, 21.8, 21.8, 17.8, 17.7, 17.6, -4.9, -5.1, -5.7, -5.8, -5.8. MALDI-HRMS *m/z* calcd. for $C_{38}H_{56}N_7O_6Si_2$ $[M+H]^+$ 762.38251, found 762.38260 (Δm = 0.00009, error 0.1ppm).

6-*N*-{1-[(7-Dimethylamino)-3-nitrodibenzo[*b,d*]furan-2-yl]ethyl}-2′-deoxyadenosin (20): 0.65 g (0.85 mmol, 1.0 eq.) **19** were dissolved in 15 ml dry THF and 2.6 ml (2.55 mmol, 3.0 eq.) of 1M TBAF/THF solution added dropwise. After stirring for 2 h the solvent was removed under reduced pressure and the residue purified via column chromatography (CH_2Cl_2:MeOH = 20:1) to obtain **20** as a red foam (0.44 g, 98%). R_f = (CH_2Cl_2:MeOH = 15:1) 0.35. ^{1}H-NMR (500 MHz, DMSO-d_6) δ [ppm] = 8.58 (m, 1H), 8.38-8.04 (m, 4H), 7.79 (d, 1H, *J* = 9 Hz), 6.91 (s, 1H), 6.86 (d, 1H, *J* = 9 Hz), 6.42-5.95 (m, 2H), 5.26 (s, 1H), 5.12-5.10 (m, 1H), 4.37 (m, 1H), 3.83 (m, 1H), 3.58-3.56 (m, 1H), 3.50-3.45 (m, 1H), 3.04 (s, 6H), 2.67 (m, 1H), 2.20 (m, 1H), 1.72 (d, 3H, *J* = 7 Hz). ^{13}C-NMR (125.8 MHz, DMSO-d_6) δ [ppm] = 160.4, 152.3, 152.1, 152.1, 148.4, 145.0, 139.7, 129.4, 122.0, 110.1, 109.8, 106.9, 93.6, 88.0, 87.9, 84.0, 70.9, 70.8, 61.8, 61.7, 45.2, 40.2, 23.3, 22.0. MALDI-HRMS *m/z* calcd. for $C_{26}H_{28}N_7O_6$ $[M+H]^+$ 534.20956, found 534.20829 (Δm = 0.00127, error 2.4ppm).

5'-*O*-(4,4'-Dimethoxytrityl)-6-*N*-{1-[(7-dimethylamino)-3-nitrodibenzo[*b*,*d*]furan-2-yl]ethyl}-2'-deoxyadenosin (21): 170 mg (0.32 mmol, 1.0 eq.) **20** were coevaporated twice with 10 ml dry pyridine and then dissolved. 0.14 g (0.42 mmol, 1.3 eq.) DMTrCl were added in portions and stirred for 2 h at room temperature. Afterwards a further small amount of DMTrCl was added (0.07 g, 0.21 mmol 0.65 eq.) to the solution and stirred overnight. The solvent was removed under reduced pressure, the residue redissolved in CH_2Cl_2 and washed with 5% citric acid and then twice with saturated $NaHCO_3$-solution. The aqueous layers were extracted twice with CH_2Cl_2, dried over Na_2SO_4 and the solvent removed under reduced pressure. Purification was performed via column chromatography (CH_2Cl_2:MeOH = 100:1, column packed with 1% NEt_3) to obtain **21** as a red solid (0.2 g, 51%). R_f = (CH_2Cl_2:MeOH = 20:1) 0.38. ^{1}H-NMR (500 MHz, DMSO-d$_6$) δ [ppm] = 8.56 (m, 1H), 8.32-7.96 (m, 4H), 7.78-7.74 (m, 1H), 7.29-7.27 (m, 2H), 7.18-7.05 (m, 7H), 6.90 (s, 1H), 6.85-6.82 (m, 1H), 6.79-6.75 (m, 4H), 6.32-5.95 (m, 2H), 5.33 (m, 1H), 4.44-4.36 (m, 1H), 3.95 (m, 1H), 3.70 (s, 6H), 3.14-3.13 (m, 2H), 3.02 (s, 6H), 2.82-2.79 (m, 1H), 2.27 (m, 1H), 1.72 (d, 3H, *J* = 7 Hz). ^{13}C-NMR (125.8 MHz, DMSO-d$_6$) δ [ppm] = 160.3, 158.0, 157.9, 152.3, 152.1, 145.0, 145.0, 144.8, 135.7, 135.6, 135.5, 135.3, 129.7, 129.6, 129.6, 129.4, 127.6, 127.6, 126.5, 121.9, 113.0, 110.1, 109.8, 93.6, 85.8, 85.4, 83.3, 70.6, 64.0, 45.0, 40.2, 22.0, 11.1. MALDI-HRMS *m/z* calcd. for $C_{47}H_{46}N_7O_8$ $[M+H]^+$ 836.34024, found 836.34083 (Δm = 0.00059, error 0.7ppm).

3'-*O*-(2-cyanoethoxy-*N*,*N*'-diisopropylamino)phosphine-5'-*O*-(4,4'-dimethoxytrityl)- 6-*N*-{1-[(7-dimethylamino)-3-nitrodibenzo[*b*,*d*]furan-2-yl]ethyl}-2'-deoxyadenosin (11): 256 mg (0.31 mmol, 1.0 eq.) **21** and 0.3 ml (1.53 mmol, 5.0 eq.) DIPEA were dissolved in 2.5 ml dry CH_2Cl_2 and stirred for 20 min at room temperature before 0.1 ml (0.61 mmol, 2.0 eq.) 2-cyanoethyl-*N*,*N*'-diisopropylchlorophosphoramidite were added. After 2 h the solution was diluted with CH_2Cl_2 to a total volume of 50 ml, washed twice with saturated $NaHCO_3$-solution and the aqueous layers were extracted twice with CH_2Cl_2. The combined organic layers were dried over Na_2SO_4 and the solvent removed under reduced pressure. The crude product was purified via column chromatography (cyclohexane:EtOAc = 2:1 → 1:1 → 1:2) and precipitation with *n*-hexane from acetone. **11** was obtained as a red foam (174 mg, 55%). R_f = (cyclohexane:EtOAc = 1:1) 0.29 diastereomer 1, 0.23 diastereomer 2. ^{1}H-NMR (500 MHz, DMSO-d$_6$) δ [ppm] = 8.58-8.57 (m, 1H), 8.32-8.10 (m, 3H), 8.02-7.93 (m, 1H), 7.78-7.76 (m, 1H), 7.30-7.27 (m, 2H), 7.18-7.01 (m, 7H), 6.90 (s, 1H), 6.84 (d, 1H, *J* = 8 Hz), 6.79-6.74 (m, 4H), 6.34-5.94 (m, 2H), 4.73-4.67 (m, 1H), 4.10-4.06 (m, 1H), 3.71-3.65 (m, 7H), 3.65-3.62 (m, 1H), 3.54-3.49 (m, 2H), 3.22-3.13 (m, 2H), 3.02 (m, 7H), 2.74 (t, 1H, *J* = 6 Hz), 2.65 (t, 1H, *J* = 6 Hz), 2.41 (m, 1H), 1.72 (d, 3H, *J* = 7 Hz), 1.12-1.07 (m, 9H), 1.01 (d, 3H, *J* = 7 Hz). ^{13}C-NMR (125.8 MHz, DMSO-d$_6$) δ [ppm] = 160.3, 157.9, 157.9, 157.9, 153.2, 152.2, 152.0, 148.2, 144.9, 144.9, 144.6, 140.1, 139.8, 136.4, 135.5, 135.4, 135.3, 135.2, 135.2, 135.1, 129.6, 129.6, 129.5, 129.4, 129.3, 127.5, 127.5, 127.4, 126.4, 121.8, 119.7, 119.6, 118.9, 118.7, 117.2, 112.9, 110.0, 109.7, 106.8, 93.5, 85.4, 85.4, 84.7, 84.4, 83.6, 73.2, 73.1, 72.5, 63.4, 63.2, 58.4, 58.4, 58.3, 58.2, 54.9, 44.9, 42.5, 42.5, 42.4, 42.4, 40.1, 37.2, 24.3, 24.2, 24.2, 24.2, 24.1, 24.1, 24.0, 22.5, 21.9, 19.8, 19.7, 19.6. ^{31}P-NMR (202. 5 MHz, DMSO-d$_6$) δ [ppm] = 147.6, 147.6, 147.0, 147.0. ESI-MS *m/z* calcd. For $C_{56}H_{63}N_9O_9P$ $[M+H]^+$ 1036.45, found 1036.57.

2. Additional 1P-Absorption Data

Figure S1 shows concentration-dependent 1P-absorption spectra of DMA-NDBF-OH (**2**) in DMSO and proves that the maximum at 424 nm does not shift between a concentration of 1000 µM and 10 µM.

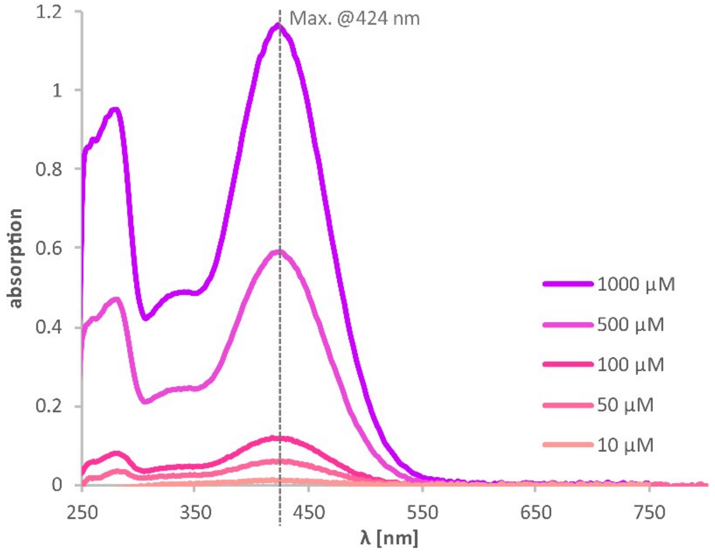

Fig. S1 1P-absorption spectra of DMA-NDBF-OH (**2**) in DMSO at different concentrations.

In analogy to the study by Riguet and Bochet[3] we investigated the protonation-dependent and solvent-dependent behaviour of the absorption spectra. These are shown in Figures S2, S3 and S5. Figure S4 shows the behaviour after irradiation at 455 nm in a buffer at pH 2.

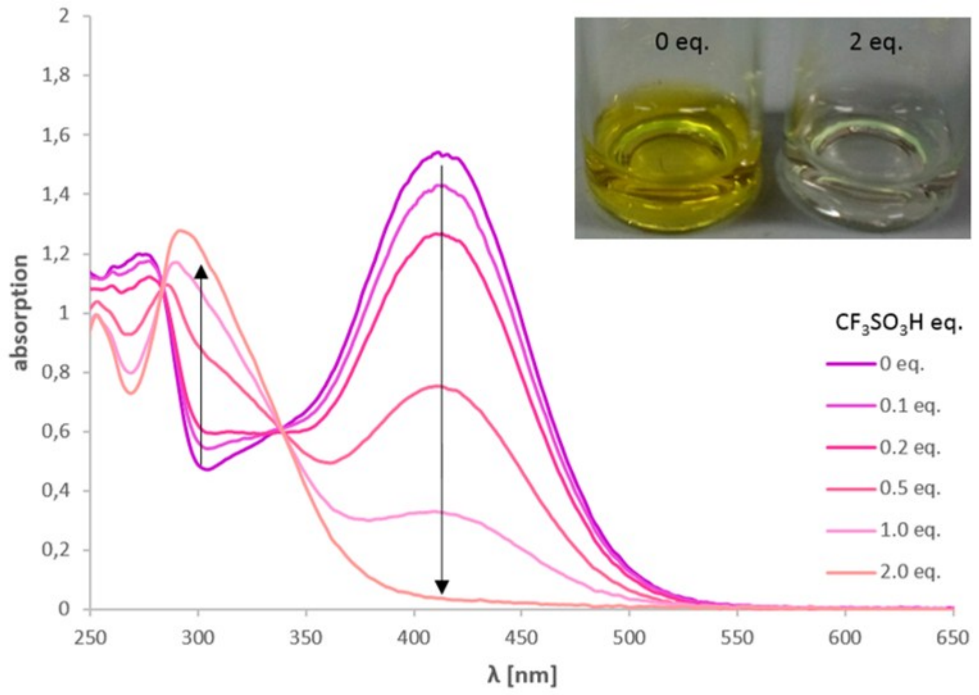

Fig. S2 1P-absorption spectra of DMA-NDBF-OH (**2**) in MeCN in presence of 0-2 eq. trifluoromethanesulfonic acid.

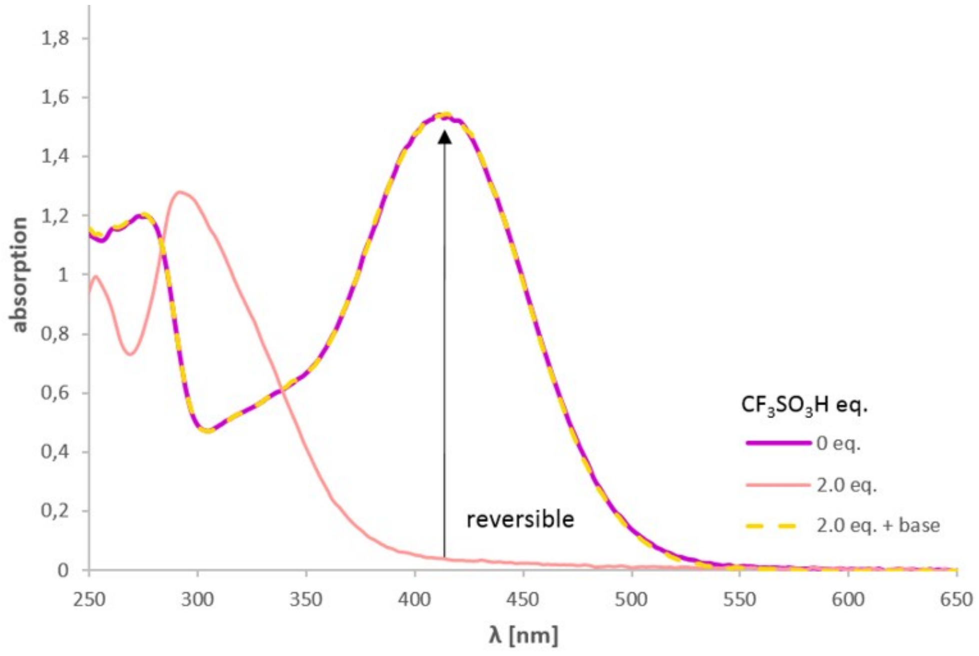

Fig. S3 1P-absorption spectra of DMA-NDBF-OH (**2**) in MeCN without additive (purple), acidified with 2.0 eq. acid (pink) and neutralised (yellow dotted).

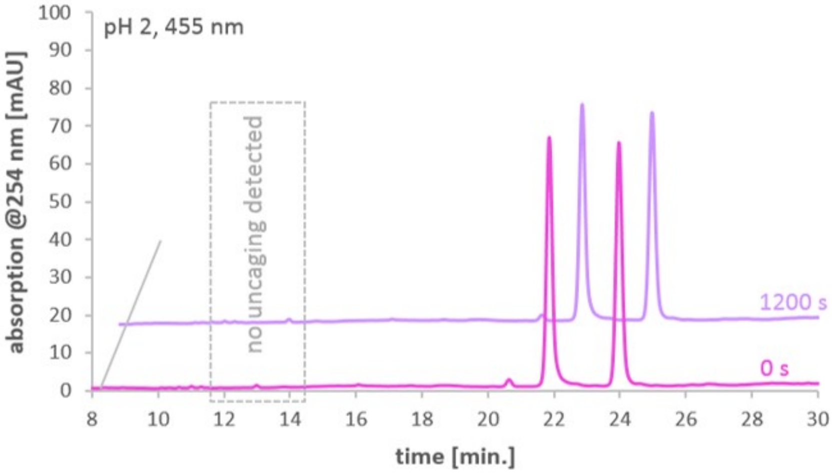

Fig. S4 HPLC analysis of **DNA2** after irradiation at 455 nm in citric acid buffer (pH 2).

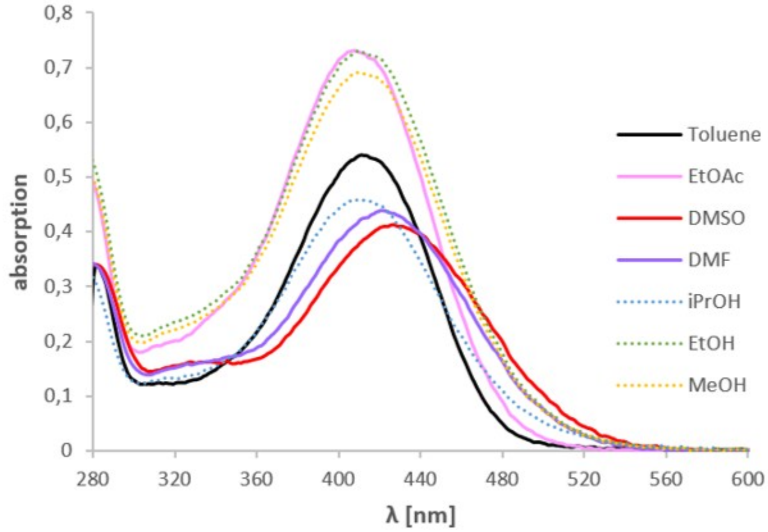

Fig. S5 1P-absorption spectra of DMA-NDBF-OH (**2**) (c = 200 µM) in various solvents.

3. Emission spectra

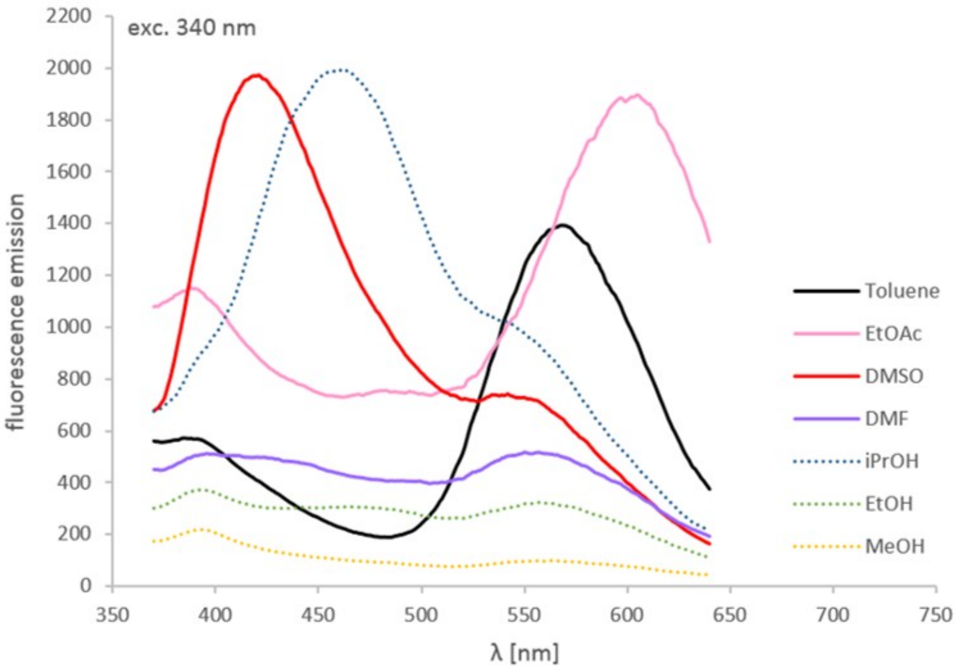

Fig. S6 Fluorescence emission spectra of DMA-NDBF-OH (**2**) in toluene, EtOAC, DMSO, DMF, iPrOH, EtOH, MeOH with $\lambda_{exc.}$ = 340 nm.

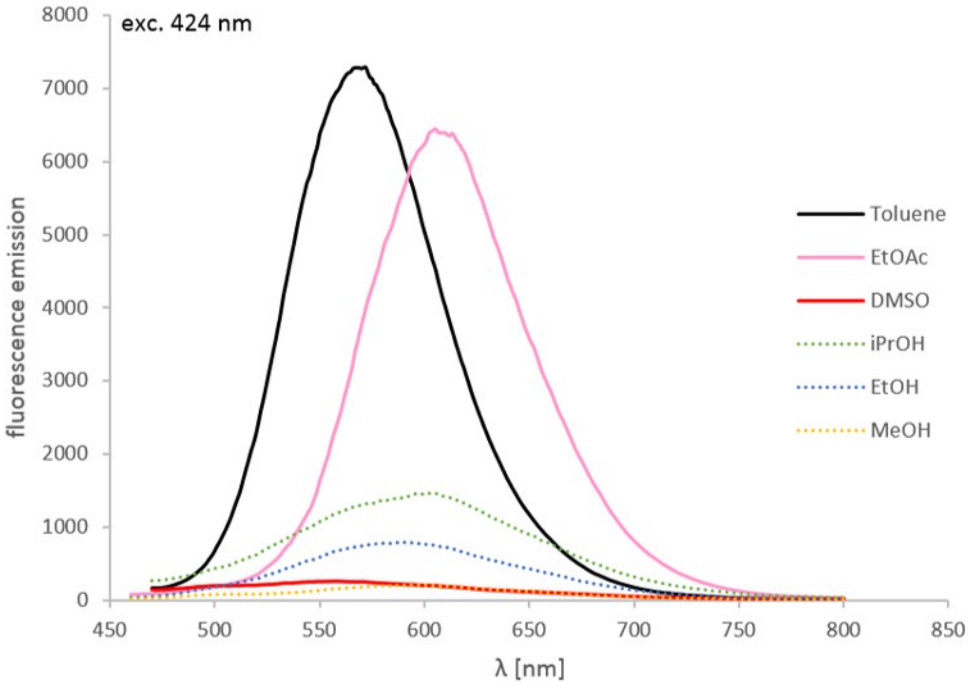

Fig. S7 Fluorescence emission spectra of DMA-NDBF-OH (**2**) in toluene, EtOAC, DMSO, iPrOH, EtOH, MeOH with $\lambda_{exc.}$ = 424 nm.

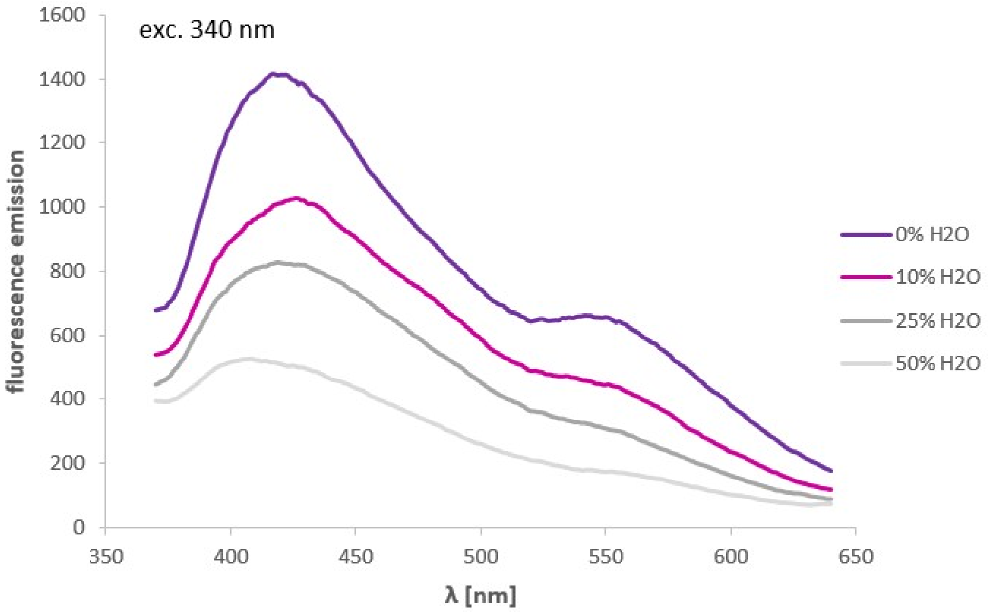

Fig. S8 Fluorescence emission spectra of DMA-NDBF-OH (**2**) in DMSO and 0-50% water.

The fluorescence quantum yield for DMA-NDBF-OH was measured in DMSO: Φ_{fl} = 0,0099 (Rhodamine B reference).

4. Wavelength-dependence of the quantum yield for the uncaging of DNA 1 and DNA2

The 1P-quantum yield for uncaging of **DNA2** with $dA^{DMA\text{-}NDBF}$ was found to be 1.10% at 340 nm and 0.05% at 420 nm while no conversion was detected at wavelengths >455 nm. Figure S9 shows a deconvolution of the absorbance spectrum of compound **2** (Figure 2). The original absorption spectrum is shown in red. Fitting a Gaussian curve around the maximum at 424 nm onto the absorbance spectrum yielded the curve shown in purple dots and a residual spectrum shown in grey in Figure S9. Fitting a Gaussian curve around 336 nm onto this residual spectrum yielded the curve shown in dashed purple lines. The sum of both purple curves is drawn in black in Figure S9. Thus, the 1P-quantum yield for the uncaging of **DNA2** depends on respective proportional contribution of the transition with maximum around 336 nm.

The 1P-quantum yields for **DNA1** with dA^{NDBF} were determined to be 24.05% with irradiation at 340 nm and 13.6% with irradiation at 420 nm.

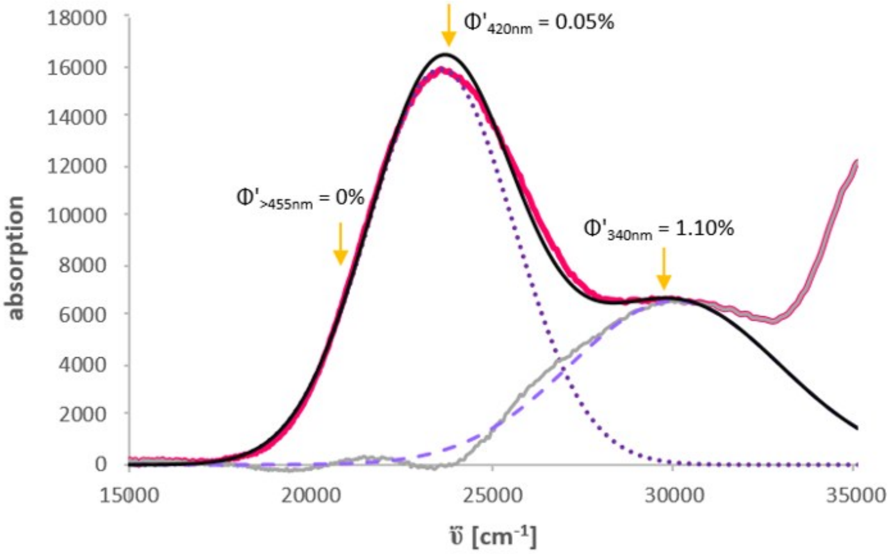

Fig. S9 Spectral unmixing of the different states in the absorption spectrum of **DMA-NDBF-OH** (**2**) and the 1P-quantum yields for the uncaging of **DNA2**. The absorption is plotted against the wavenumber.

5. 2P-Excitation Experiments with NDBF-OH (1) and DMA-NDBF-OH (2)

Details of the setup for two-photon excitation spectroscopy have been described previously.(Gacek *et al.*[4]). Briefly, a Chameleon Ultra II, 80 MHz laser system (APE Berlin and Coherent Inc.) was used to generate pulsed laser excitation of wavelengths in the range from 700-1060 nm. Using a 1000 nm reflection/705 nm transition dichroic mirror (AHF T700spxr-1500) the expanded laser beam was reflected into the back-aperture of a water immersion IR microscope objective (UPlanApo/IR 60×1.20 W) in a confocal microscope setup (microscope body IX71, Olympus). To ensure a constant two-photon excitation power throughout the entire spectral range, a calibrated power meter head (coherent LM-2 VIS) was installed at a fixed point of 5 cm above the microscope objective. A linear variable neutral density filter (NDL-10C-2, Thorlabs) was used before the microscope body to keep the two-photon excitation power at 1 mW at the power meter head. To effectively suppress any IR excitation light in the detection path two IR-block filters (AHF HC770/SP) were used. The fluorescence after two-photon excitation was detected by an electron multiplying charge coupled device (EMCCD) camera (iXonEM + 897 back-illuminated, Andor Technology). The emission spots recorded with the camera were integrated for each excitation wavelength and corrected for background noise.

6. DNA-Synthesis

DNA synthesis was carried out on an ABI392 Synthesizer from *Applied Biosystems*. The CPG solid support were purchased from *Link Technologies* for 0.2 µmol or 1.0 µmol scale syntheses. All strands were synthesised with standard protocols, partly under UltraMILD® conditions (*Glen Research*), and with "DMTr-On". Deprotection from solid support occurred with 32% NH_3/H_2O overnight or for 4 h (UltraMILD®) at room temperature. The evaporated samples were purified via RP-HPLC (MultoKrom® 100-5 C18, *CS-Chromatographie GmbH*) with 0.1 M triethylammonium acetate buffer (pH 7.0) and MeCN. The DMTr groups were removed with 80% acetic acid, followed by a second purification RP-HPLC run with the same gradient (5-40% MeCN in 33 min or longer with same slope). For **DNA3**, **4** and **5** a thiol modifier C6 S-S phosphoramidite from *Link Technologies* was used*,* which required further treatment with tris(2-carboxyethyl)phosphine (TCEP) and RP-HPLC purification (see 8*. Hydrogel Preparation*).

7. Mass-Spectrometric Characterisation

Table S1 Used DNA strands and their mass-spectrometric characterisation.

Strand	Cage	Sequence (5' to 3')	Mass calc. [Da]	Mass found [Da]
**DNA1**	NDBF	GCATAAA***A***^{NDBF}AAAGGTG	4897.3	4896.9
**DNA2**	DMA-NDBF	GCATAAA***A***$^{DMA\text{-}NDBF}$AAAGGTG	4940.4	4940.0
**DNA3**	NDBF	SH-$(CH_2)_6$-GC***A***^{NDBF}TAA***A***^{NDBF}TAA***A***^{NDBF}GGTG	5693.1*	5692.6*
**DNA4**	DMA-NDBF	SH-$(CH_2)_6$-GC***A***$^{DMA\text{-}NDBF}$TAA***A***$^{DMA\text{-}NDBF}$TAA***A***$^{DMA\text{-}NDBF}$GGTG	5822.2*	5821.8*
**DNA5**	NDBF	SH-$(CH_2)_6$-AG***A***^{NDBF}TAC***A***^{NDBF}GAT***A***^{NDBF}CGCA	5640.2*	5639.1*
F_1 = ATTO565 labelled**	-	F_1-NH-$(CH_2)_6$-CACCTTTATTTATGC	5152.0	5150.0
F_2 = ATTORho14 labelled	-	F_2-NH-$(CH_2)_6$-TGCGTATCTGTATCT	5491.4	5491.1
Q1= BHQ2**	-	TAAATAAAGGTG-Q_1	4270.8	4273.7
Q2 = BBQ-650III	-	ACAGATACGCA-Q_2	3997.8	3999.5

*dithiol
**purchased from *biomers.net GmbH*

ATTO565 = fluorescent dye from *ATTO-TEC GmbH* with λ_{max} = 565 nm, ATTORho14 = fluorescent dye from *ATTO-TEC GmbH* based on a rhodamine structure, BHQ-x = "Black Hole" quencher x, BBQ-x = "BlackBerry" quencher x.

8. Hydrogel Preparation

Hydrogels were prepared according to Fichte *et al.*[5] with the "slow gelling 3-D Life PVA-PEG Hydrogel Kit" from *Cellendes*. **DNA3**, **4** and **5** were treated with TCEP (50 mM in 100 mM Tris buffer, pH 7.4) for 2 h at room temperature before use to reduce disulfide bonds (thiol modifier C6 S-S phosphoramidite from *Link Technologies* was used in solid phase synthesis) and to obtain the free SH-moiety. After RP-HPLC purification (A: MeCN/B: 0.1M TEAA buffer) 75 pmol DNA in a maximum volume of 2.50 µl water were incubated for 10 min together with 1.25 µl maleimide-PVA to covalently attach the thiols in 0.63 µl CB buffer (pH 7.2). The solution was mixed with 1.87 µl PEG-Link, which consists of polyethylene glycol with a thiol at each end, to give a total volume of 6.25 µl and placed in a Nunc™ Lab-Tek™ chambered coverglass. After 30 min the droplet was surrounded with 200 µl fluorophore-quencher double-strand solution. Therefor, 225 pmol fluorophore strand and quencher 1 (1.5 eq.) or quencher 2 (2 eq.) were hybridised in PBS.
TCEP = tris(2-carboxyethyl)phosphine, TEAA = trimethylamine acetate, CB = citrate buffer by *Cellendes*, PBS = phosphate buffered saline.

9. General Orthogonal Uncaging Scheme

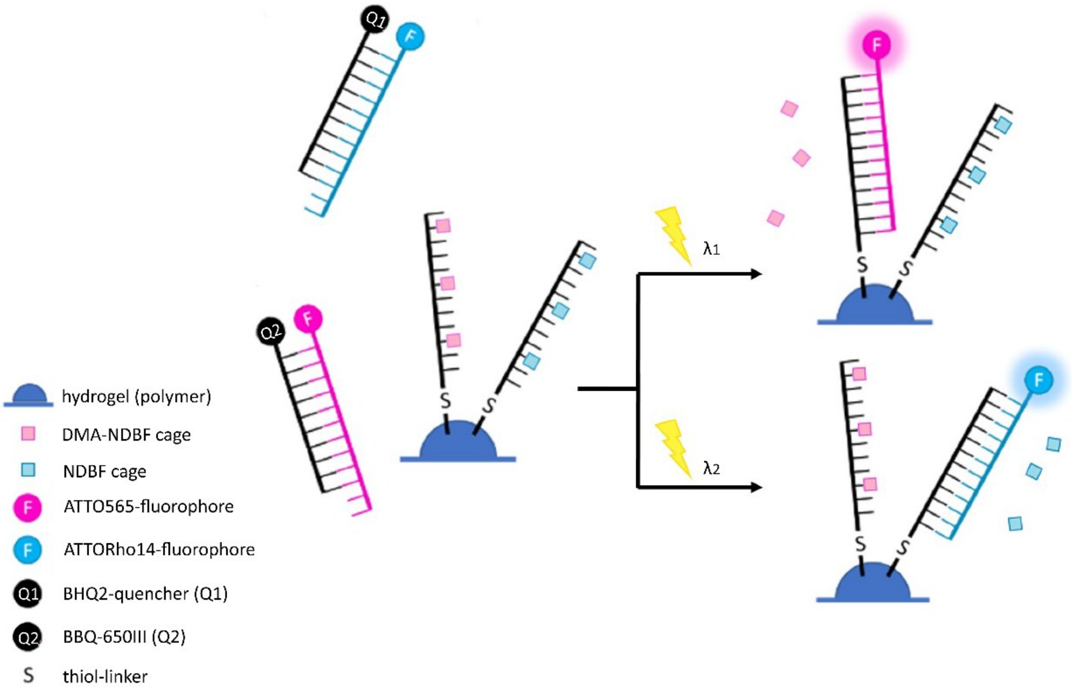

Scheme S2 Orthogonal uncaging scheme for two photolabile protecting groups which photolyse selectively irradiating at wavelengths λ_1 or λ_2.

10. Laser Setup 2 “Uncaging Setup”

For uncaging an ultrafast Ti:Sapphire Mai-Tai BB Laser from *Newport Spectra-Physics* was coupled into an confocal *Zeiss* Axio Observer.Z1 microscope with an Plan-Apochromat 63x/1.40 oil objective. The computer-controlled illumination of regions of interest with user-defined positions was realised with a UGA-40 galvanometer from *Rapp OptoElectronic*. The power was variated with a Pockels cell with installed polarizer from *Conoptics Inc.* and measured with a “LabMax-TO” powermeter and various detector heads from *Coherent*. For 1P-uncaging an “Inspire Blue” frequency doubler from *Newport Spectra-Physics* was placed in the laser beam.

11. Laser Setup 3 “Imaging Setup”

A *Zeiss* Axio Observer.Z1 LSM 710 with an 63x/1.40 oil objective was used for orthogonal imaging. The fluorescence excitation wavelengths 543 nm for ATTO565 (detection at 557-612 nm) and 633 nm for ATTORho14 (detection at 671-721 nm) were achieved with HeNe-lasers. Image processing was performed via *Zeiss'* Zen Software. For the 3D image a z-stack (Fig. S10) consisting of 88 slices with intervals of 2 µm was generated.

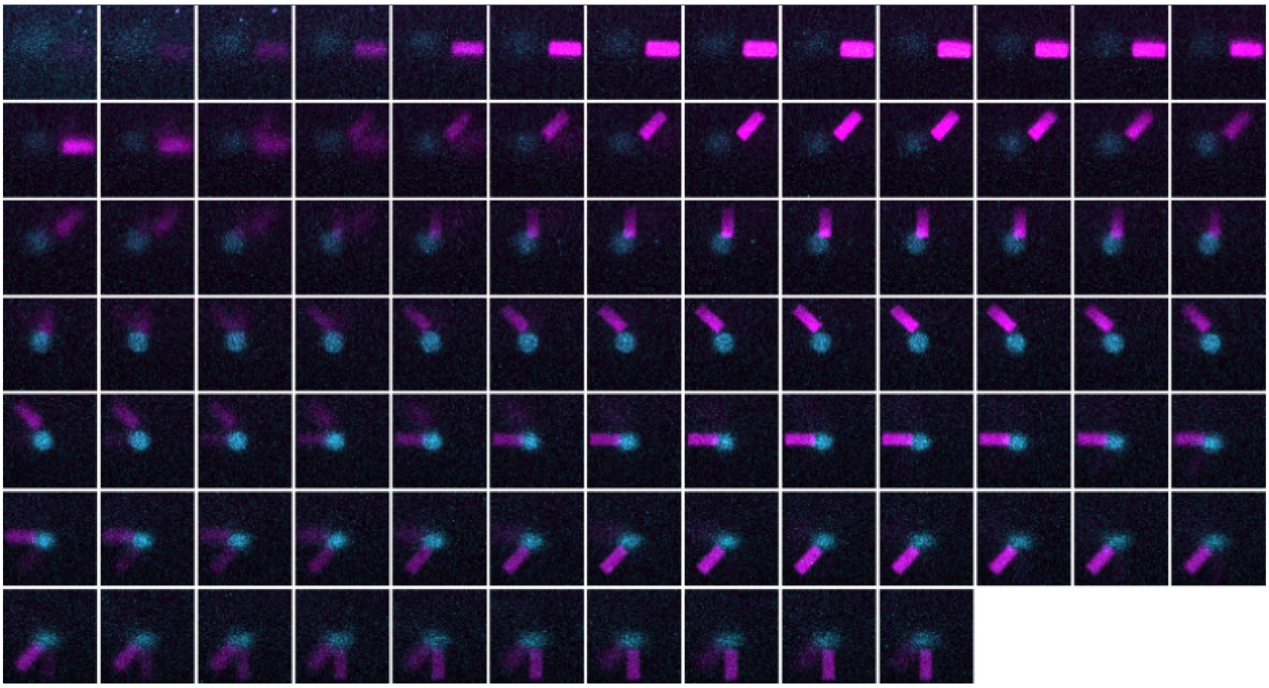

Fig. S10 Z-stack consisting of 88 images acquired with **laser setup 3**.

12. References

1 F. Schäfer, K. B. Joshi, M. A. H. Fichte, T. Mack, J. Wachtveitl, A. Heckel, *Org. Lett.* 2011, **13**, 1450-1453.

2 H. Lusic, R. Uprety, A. Deiters, *Org. Lett.* 2010, **12**, 916-919.

3 E. Riguet, C. G. Bochet, *Org. Lett.* 2007, **9**, 5453-5456.

4 D. A. Gacek, A. L. Moore, T. A. Moore, P. J. Walla, *J. Phys. Chem. B* 2017, **121**, 10055-10063.

5 M. A. H. Fichte, X. M. M. Weyel, S. Junek, F. Schäfer, C. Herbivo, M. Goeldner, A. Specht, J. Wachtveitl, A. Heckel, *Angew. Chem. Int. Ed.* 2016, **55**, 8948-8952.

13. ^{1}H-NMR spectra

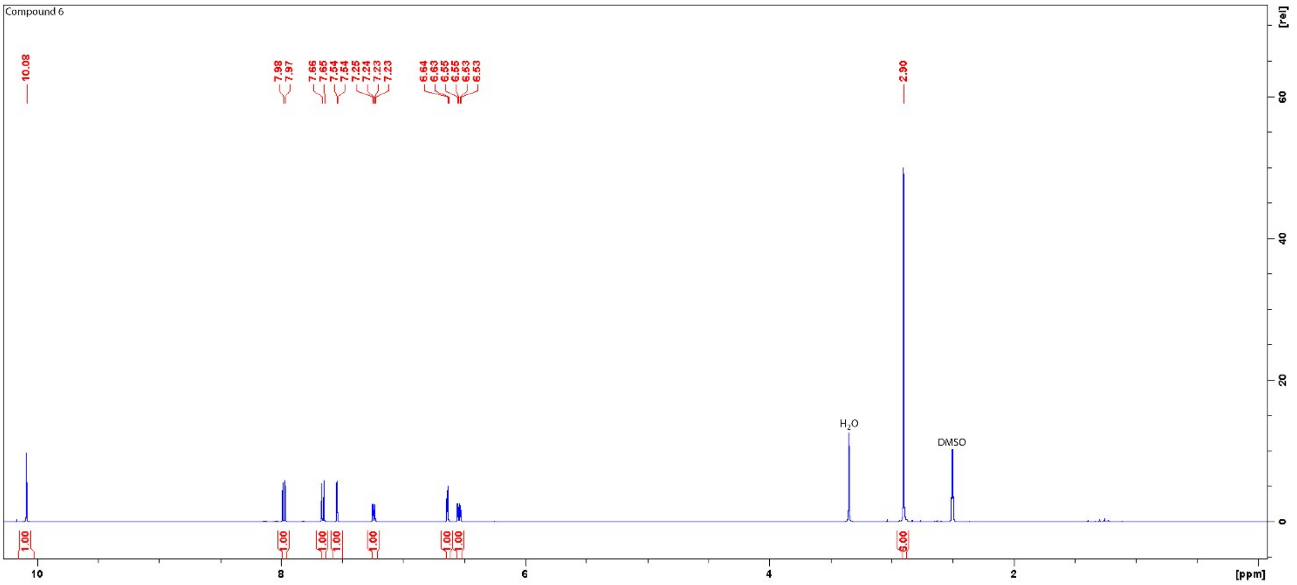

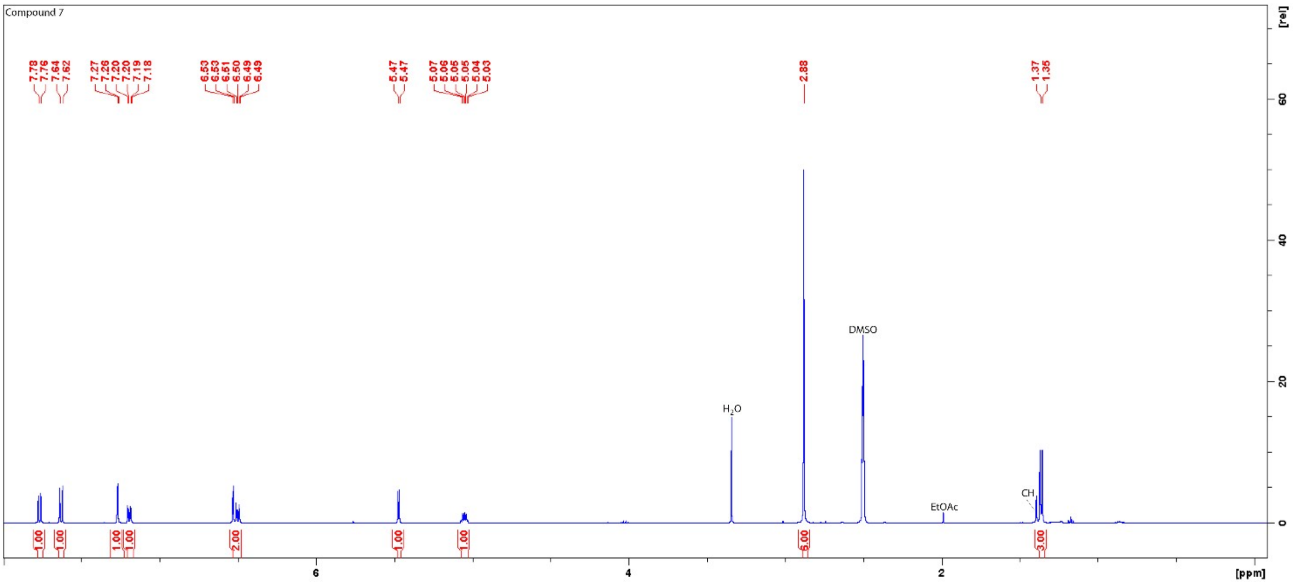

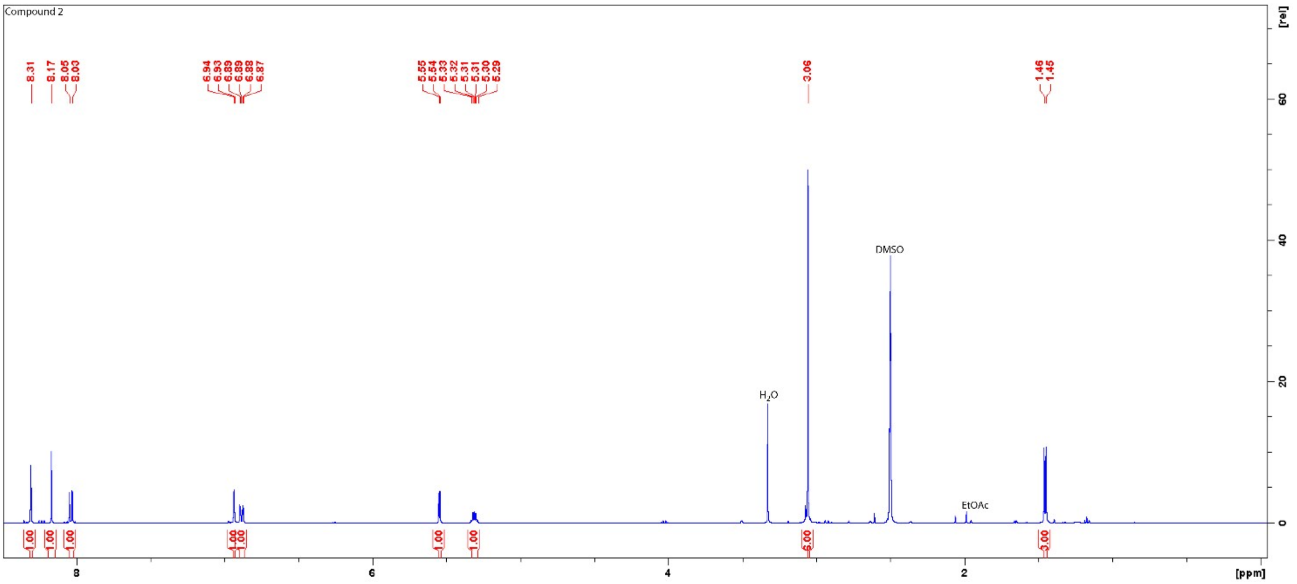

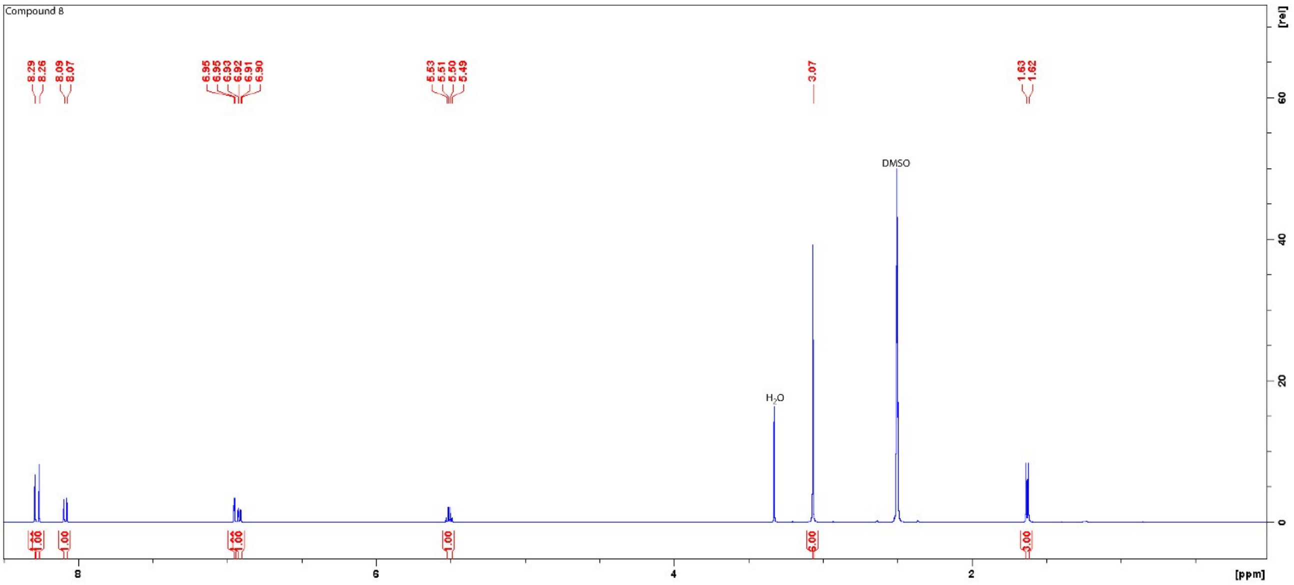
Compound 8
DMSO
H_2O
[ppm]
[rel]

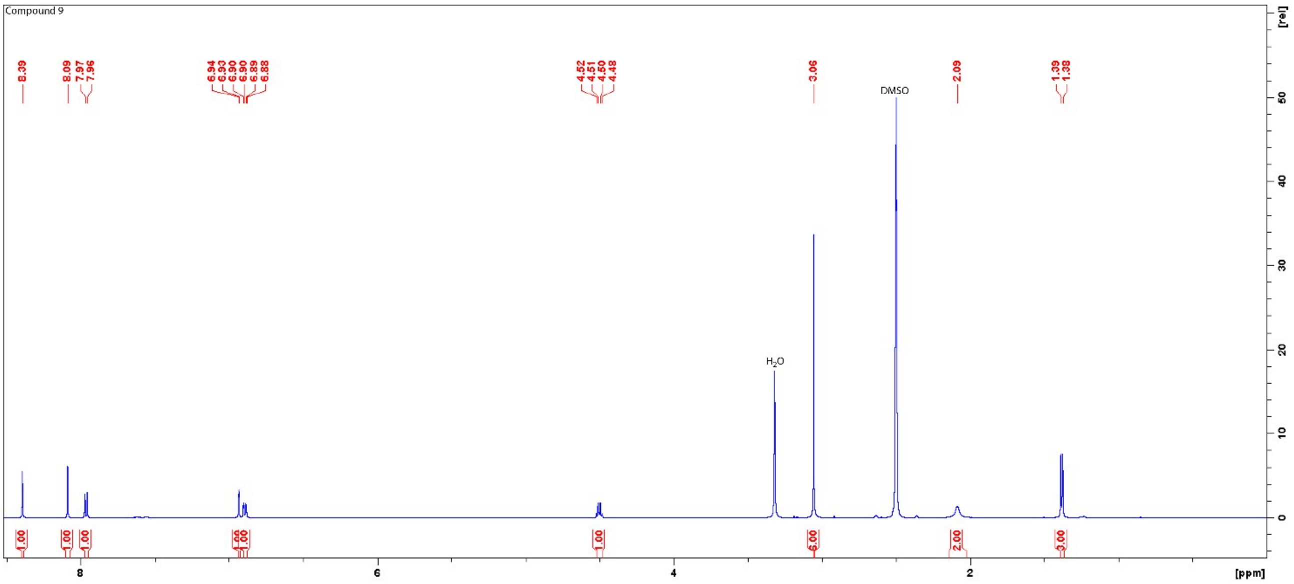
Compound 9
DMSO
H_2O
[ppm]
[rel]

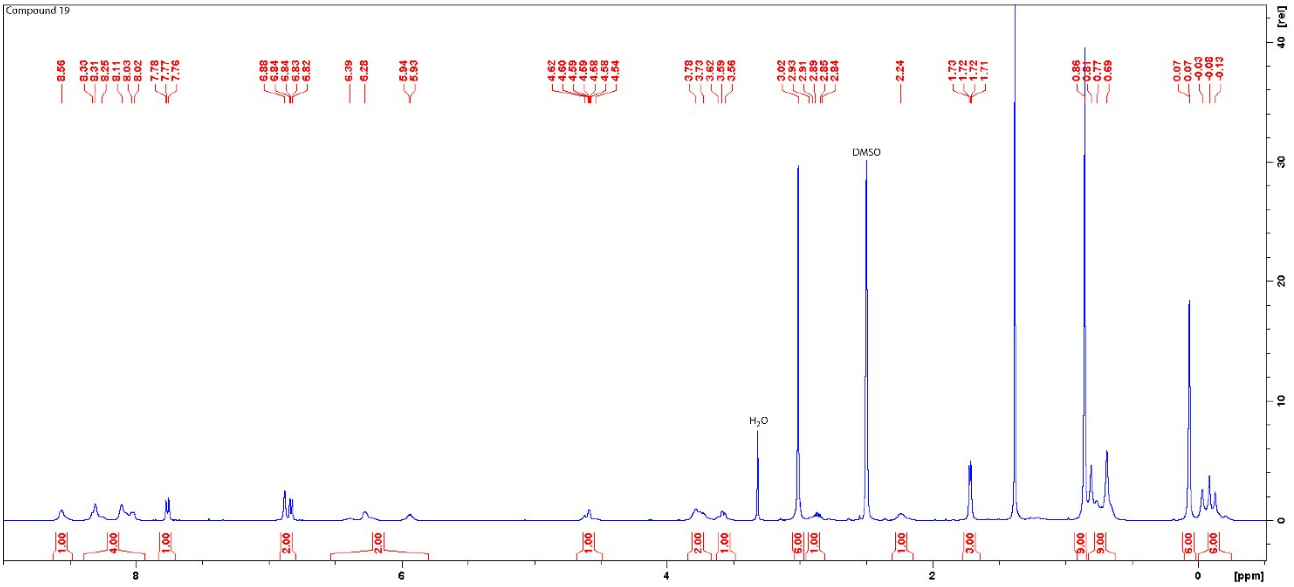
Compound 19
DMSO
H_2O
[ppm]
[rel]

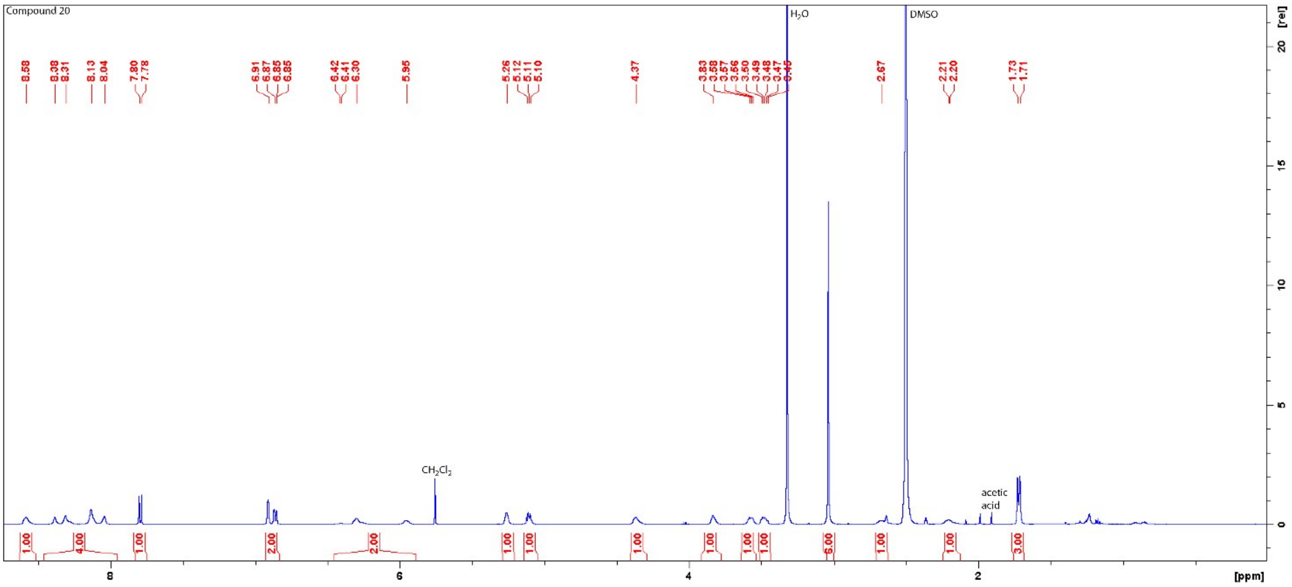
Compound 20
H2O
DMSO
CH2Cl2
acetic acid
[rel]
[ppm]

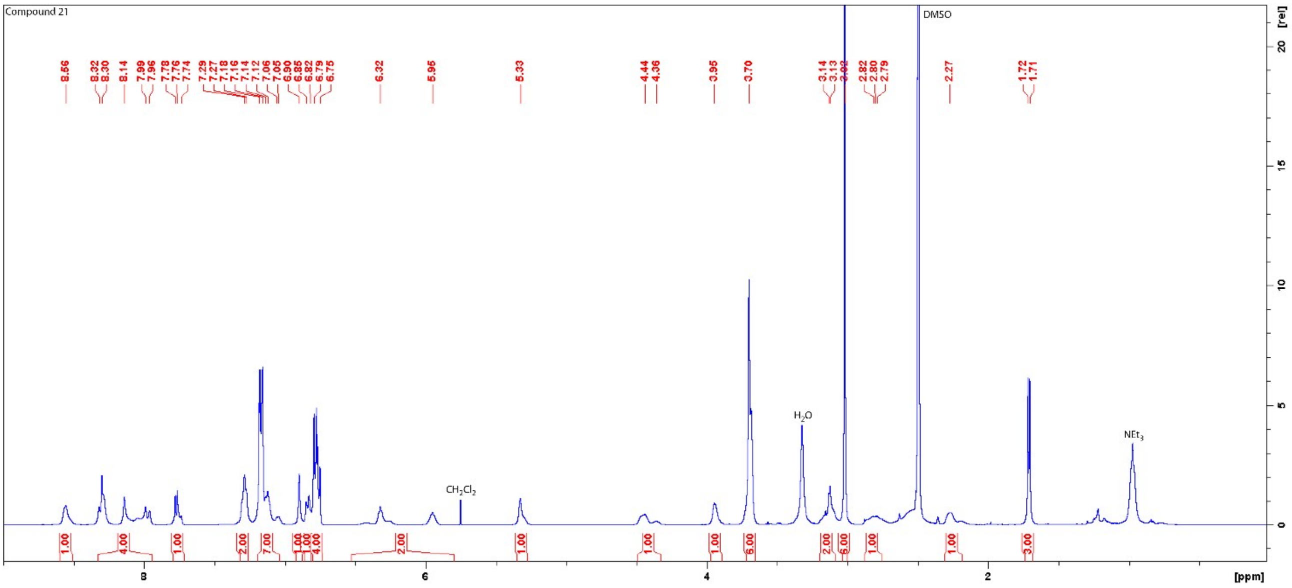
Compound 21
DMSO
H2O
CH2Cl2
NEt3
[rel]
[ppm]

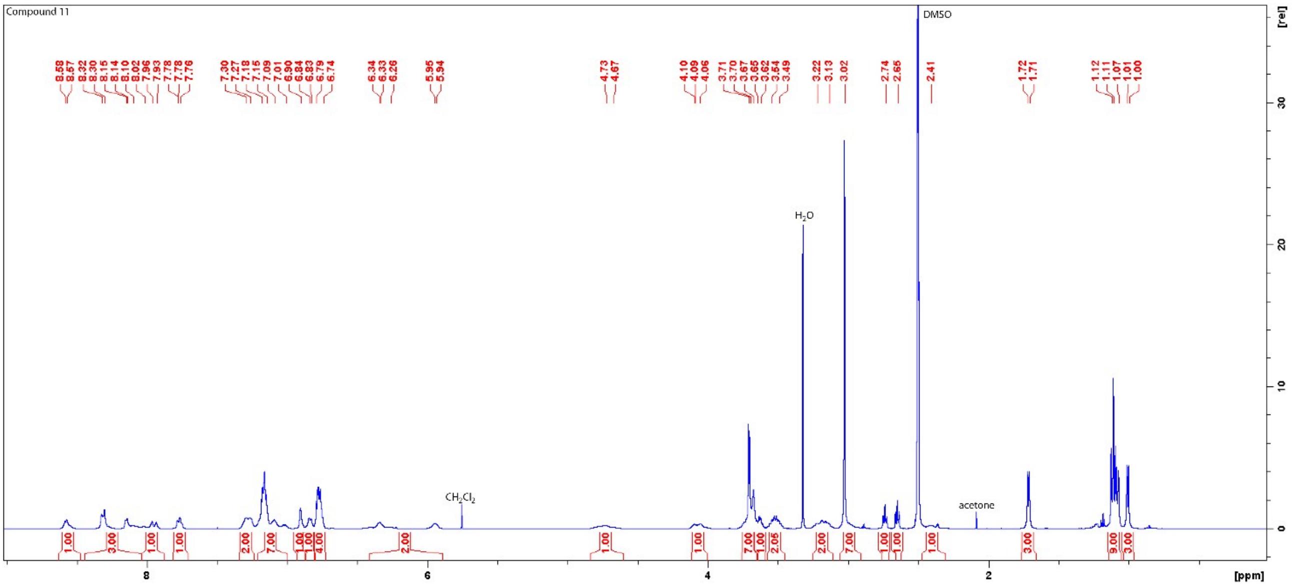
Compound 11
DMSO
H2O
CH2Cl2
acetone
[rel]
[ppm]

14. Mass spectra

DNA1 (GCATAAAA^{NDBF}AAAGGTG)

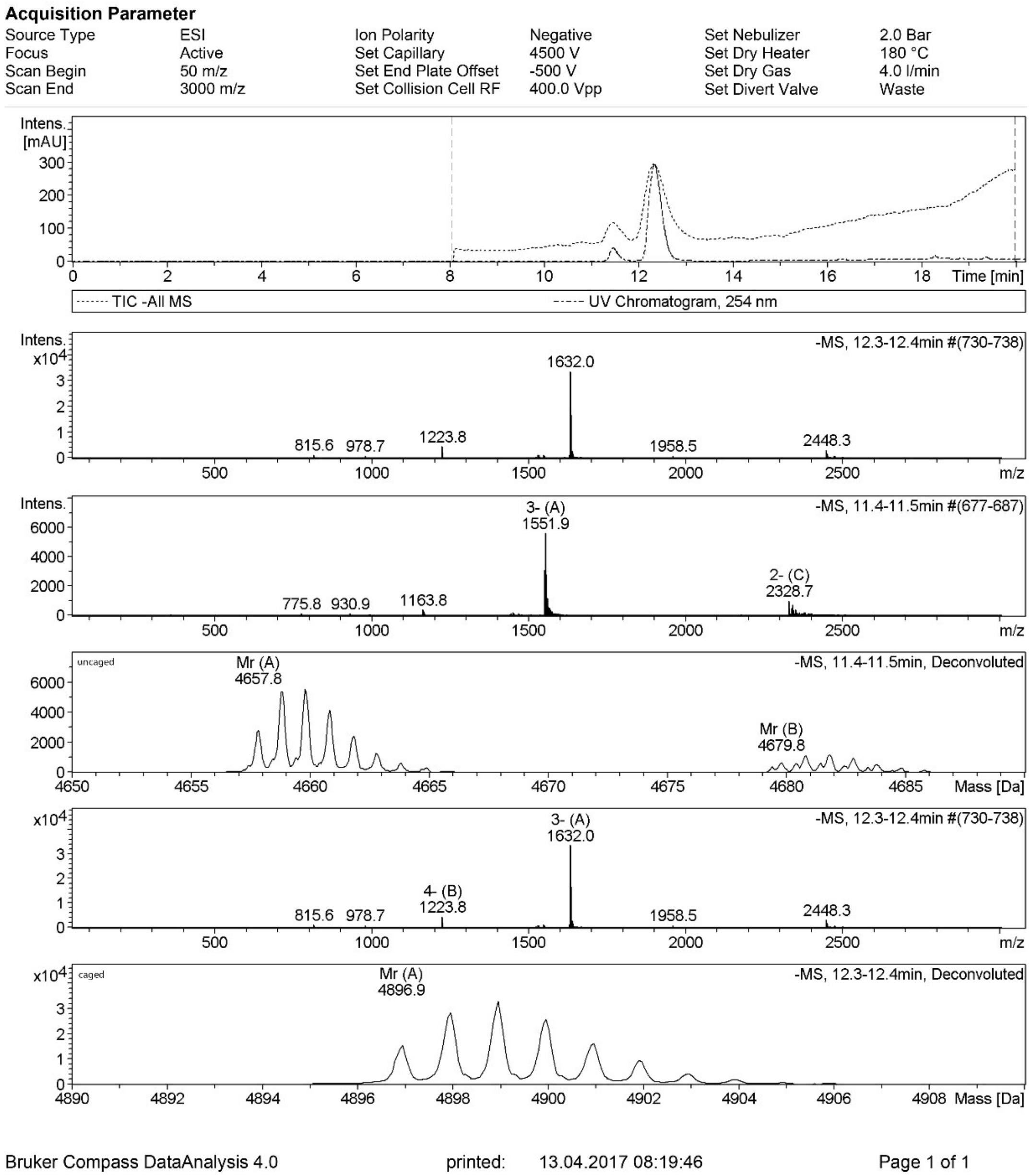

DNA2 (GCATAAAA$^{DMA\text{-}NDBF}$AAAGGTG)

Acquisition Parameter

Source Type	ESI	Ion Polarity	Negative	Set Nebulizer	2.0 Bar
Focus	Active	Set Capillary	4500 V	Set Dry Heater	180 °C
Scan Begin	50 m/z	Set End Plate Offset	-500 V	Set Dry Gas	4.0 l/min
Scan End	3000 m/z	Set Collision Cell RF	400.0 Vpp	Set Divert Valve	Waste

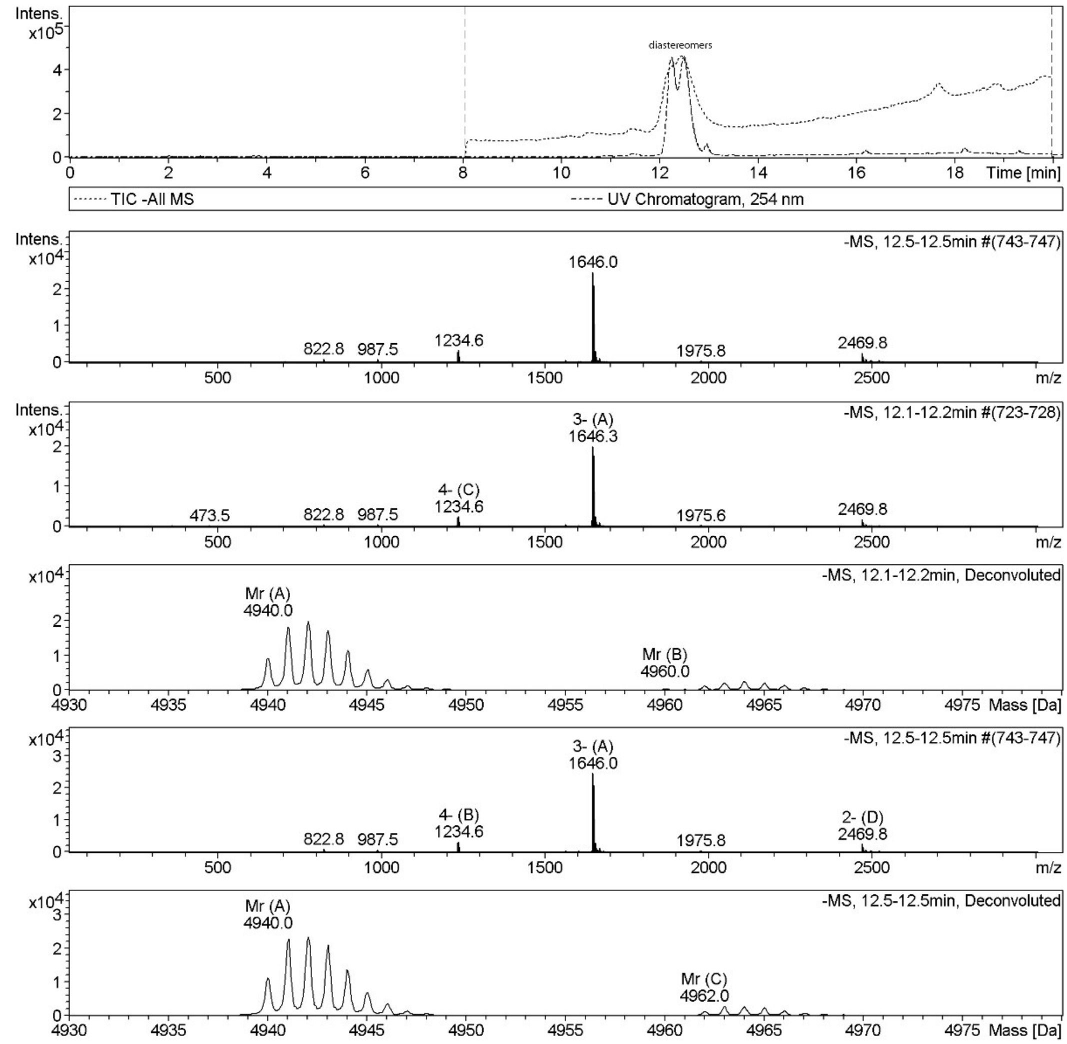

Bruker Compass DataAnalysis 4.0 printed: 02.05.2017 08:29:05 Page 1 of 1

DNA3 (SH-$(CH_2)_6$-GCA^{NDBF}TAAA^{NDBF}TAAA^{NDBF}GGTG)

Acquisition Parameter

Source Type	ESI	Ion Polarity	Negative	Set Nebulizer	2.0 Bar
Focus	Active	Set Capillary	4500 V	Set Dry Heater	180 °C
Scan Begin	50 m/z	Set End Plate Offset	-500 V	Set Dry Gas	4.0 l/min
Scan End	3000 m/z	Set Collision Cell RF	400.0 Vpp	Set Divert Valve	Waste

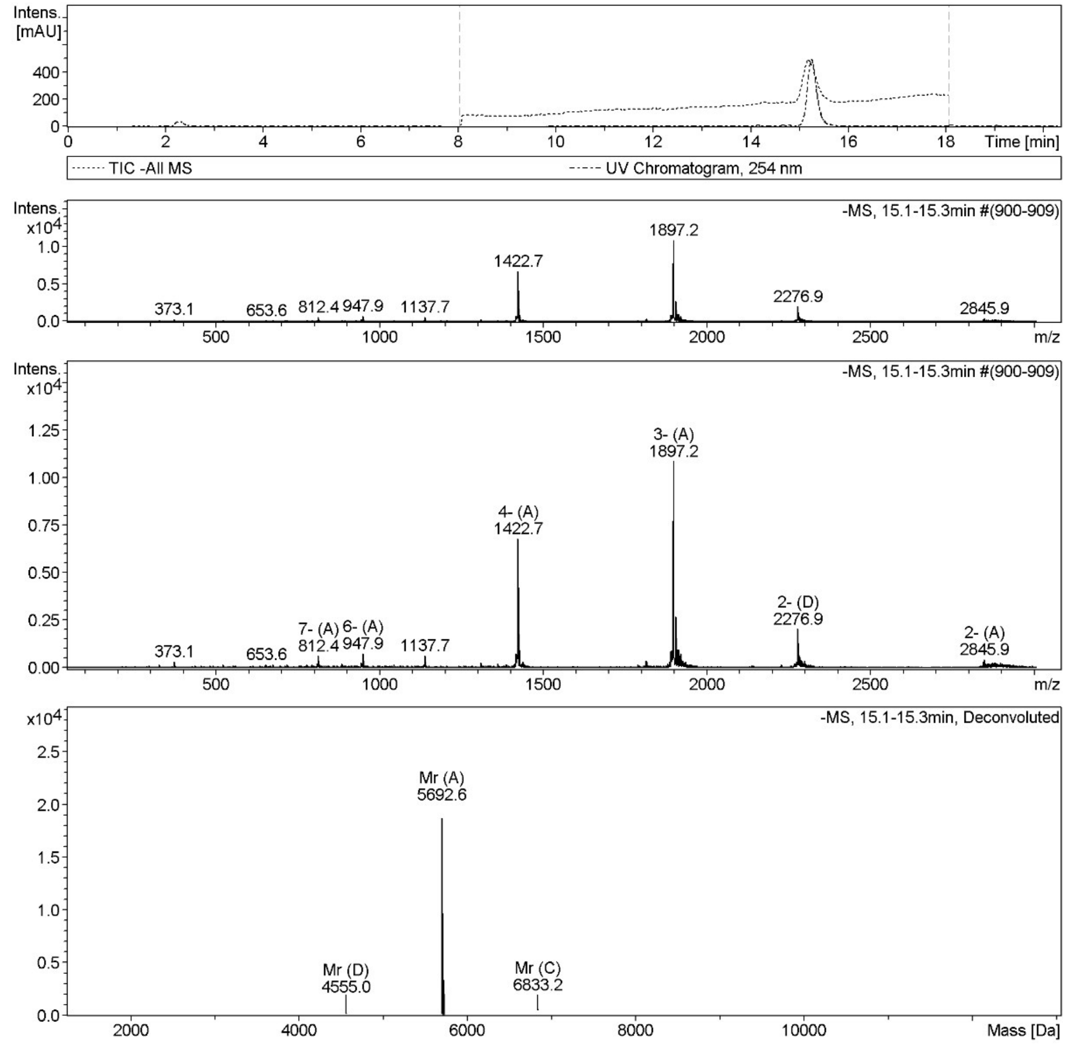

Bruker Compass DataAnalysis 4.0 printed: 15.08.2016 13:16:40 Page 1 of 1

DNA4 (SH-(CH$_2$)$_6$-GC*A*$^{DMA-NDBF}$TAA*A*$^{DMA-NDBF}$TAA*A*$^{DMA-NDBF}$GGTG)

Acquisition Parameter

Source Type	ESI	Ion Polarity	Negative	Set Nebulizer	2.0 Bar
Focus	Active	Set Capillary	4500 V	Set Dry Heater	180 °C
Scan Begin	50 m/z	Set End Plate Offset	-500 V	Set Dry Gas	4.0 l/min
Scan End	3000 m/z	Set Collision Cell RF	400.0 Vpp	Set Divert Valve	Waste

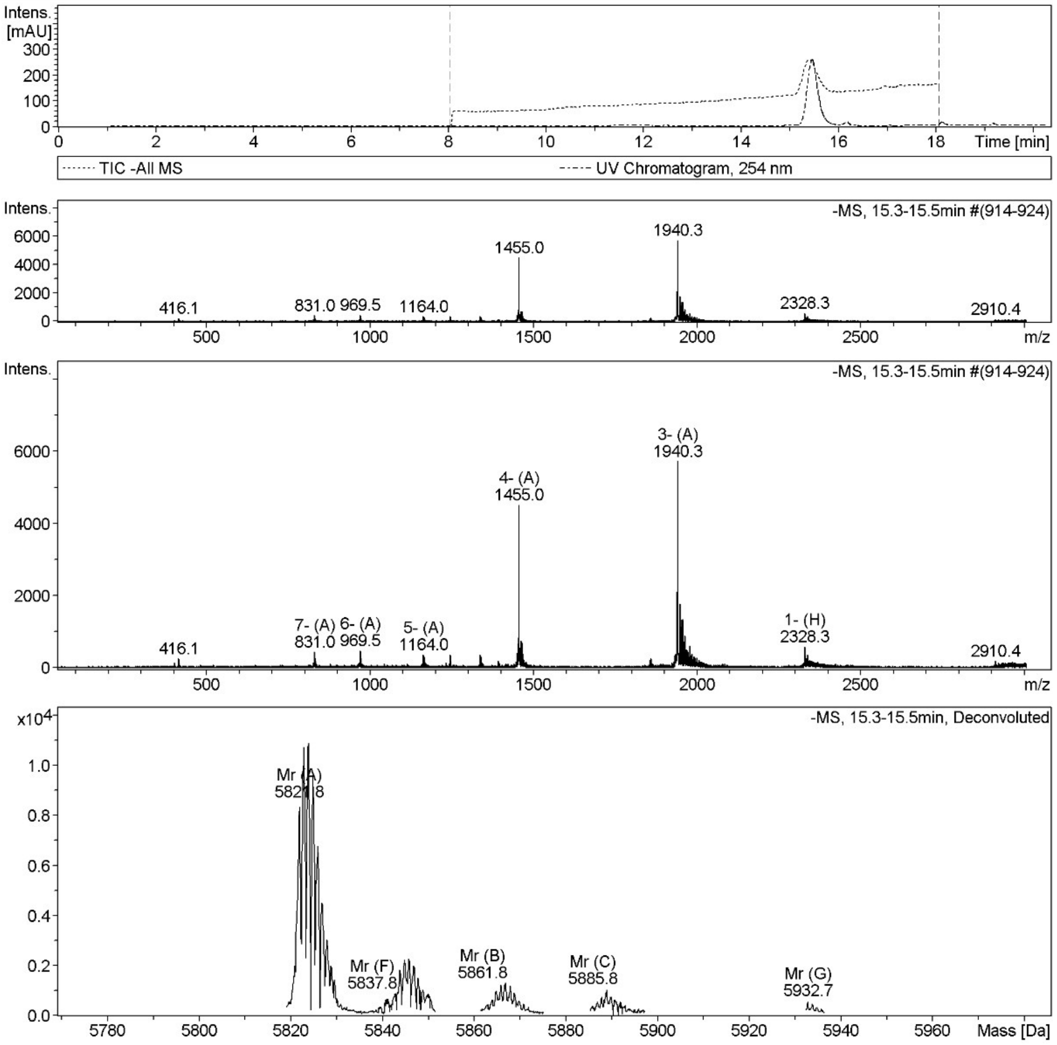

Bruker Compass DataAnalysis 4.0 printed: 01.08.2016 09:16:43 Page 1 of 1

DNA5 (SH-$(CH_2)_6$-AGA^{NDBF}TACA^{NDBF}GATA^{NDBF}CGCA)

Acquisition Parameter

Source Type	ESI	Ion Polarity	Negative	Set Nebulizer	2.0 Bar
Focus	Active	Set Capillary	4500 V	Set Dry Heater	180 °C
Scan Begin	50 m/z	Set End Plate Offset	-500 V	Set Dry Gas	4.0 l/min
Scan End	3000 m/z	Set Collision Cell RF	400.0 Vpp	Set Divert Valve	Waste

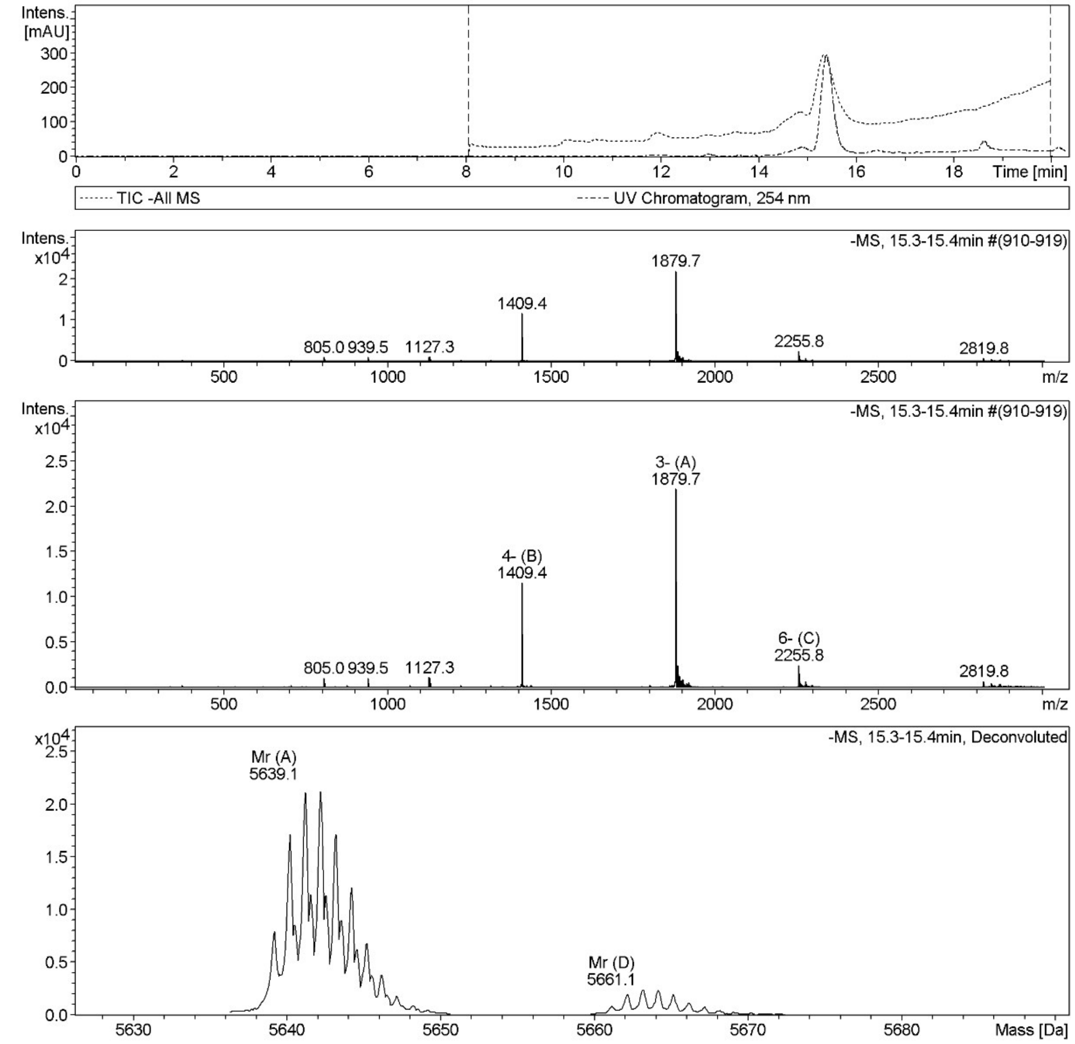

Bruker Compass DataAnalysis 4.0 printed: 13.06.2017 09:18:54 Page 1 of 1

ATTO565-NH-$(CH_2)_6$-CACCTTTATTTATGC

Acquisition Parameter

Source Type	ESI	Ion Polarity	Negative	Set Nebulizer	2.0 Bar
Focus	Active	Set Capillary	4500 V	Set Dry Heater	180 °C
Scan Begin	50 m/z	Set End Plate Offset	-500 V	Set Dry Gas	4.0 l/min
Scan End	3000 m/z	Set Collision Cell RF	400.0 Vpp	Set Divert Valve	Waste

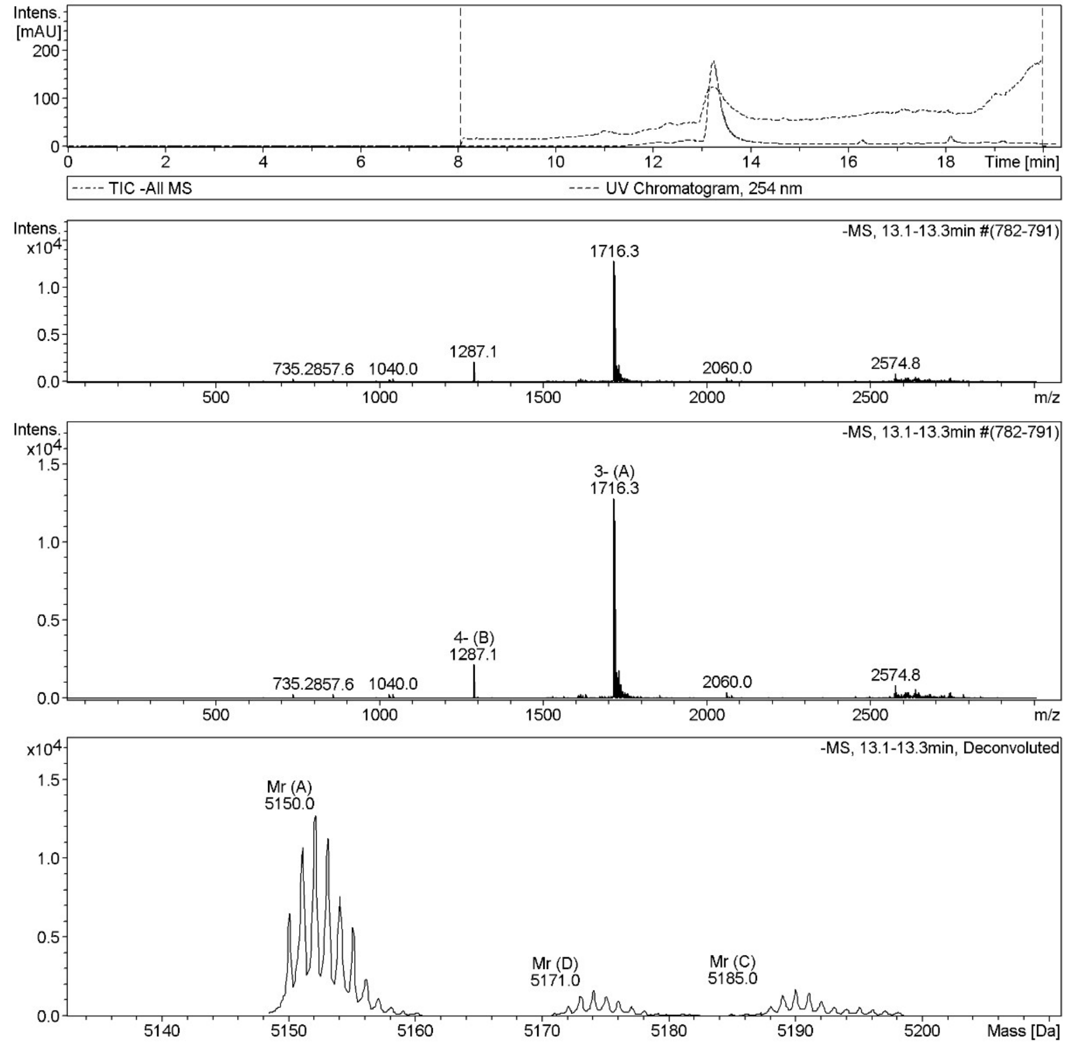

Bruker Compass DataAnalysis 4.0 printed: 19.10.2017 11:38:03 Page 1 of 1

ATTORho14-NH-$(CH_2)_6$-TGCGTATCTGTATCT

Acquisition Parameter

Source Type	ESI	Ion Polarity	Negative	Set Nebulizer	2.0 Bar
Focus	Active	Set Capillary	4500 V	Set Dry Heater	180 °C
Scan Begin	50 m/z	Set End Plate Offset	-500 V	Set Dry Gas	4.0 l/min
Scan End	3000 m/z	Set Collision Cell RF	400.0 Vpp	Set Divert Valve	Waste

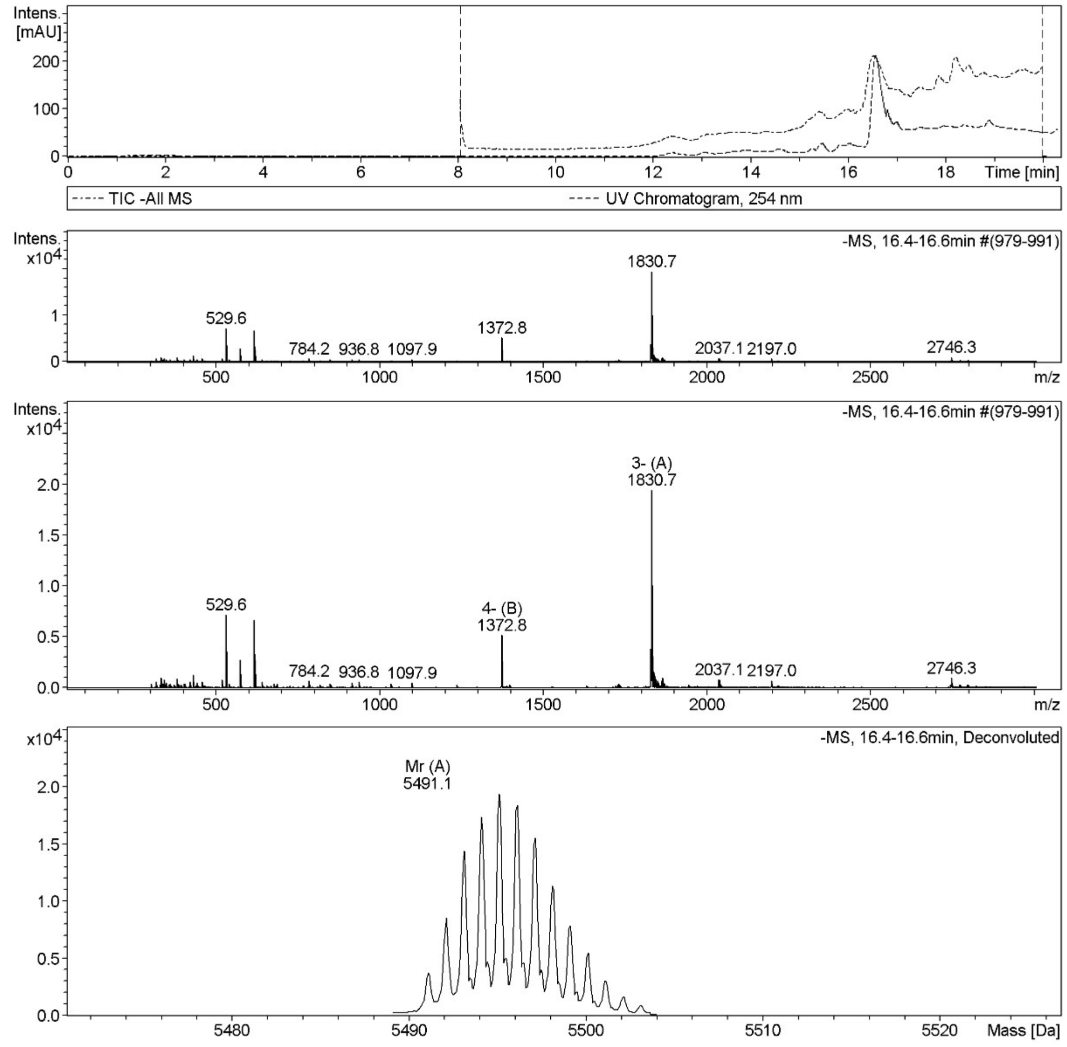

Bruker Compass DataAnalysis 4.0 printed: 13.06.2017 09:19:27 Page 1 of 1

TAAATAAAGGTG-BHQ2

Acquisition Parameter

Source Type	ESI	Ion Polarity	Negative	Set Nebulizer	2.0 Bar
Focus	Active	Set Capillary	4500 V	Set Dry Heater	180 °C
Scan Begin	50 m/z	Set End Plate Offset	-500 V	Set Dry Gas	4.0 l/min
Scan End	3000 m/z	Set Collision Cell RF	400.0 Vpp	Set Divert Valve	Waste

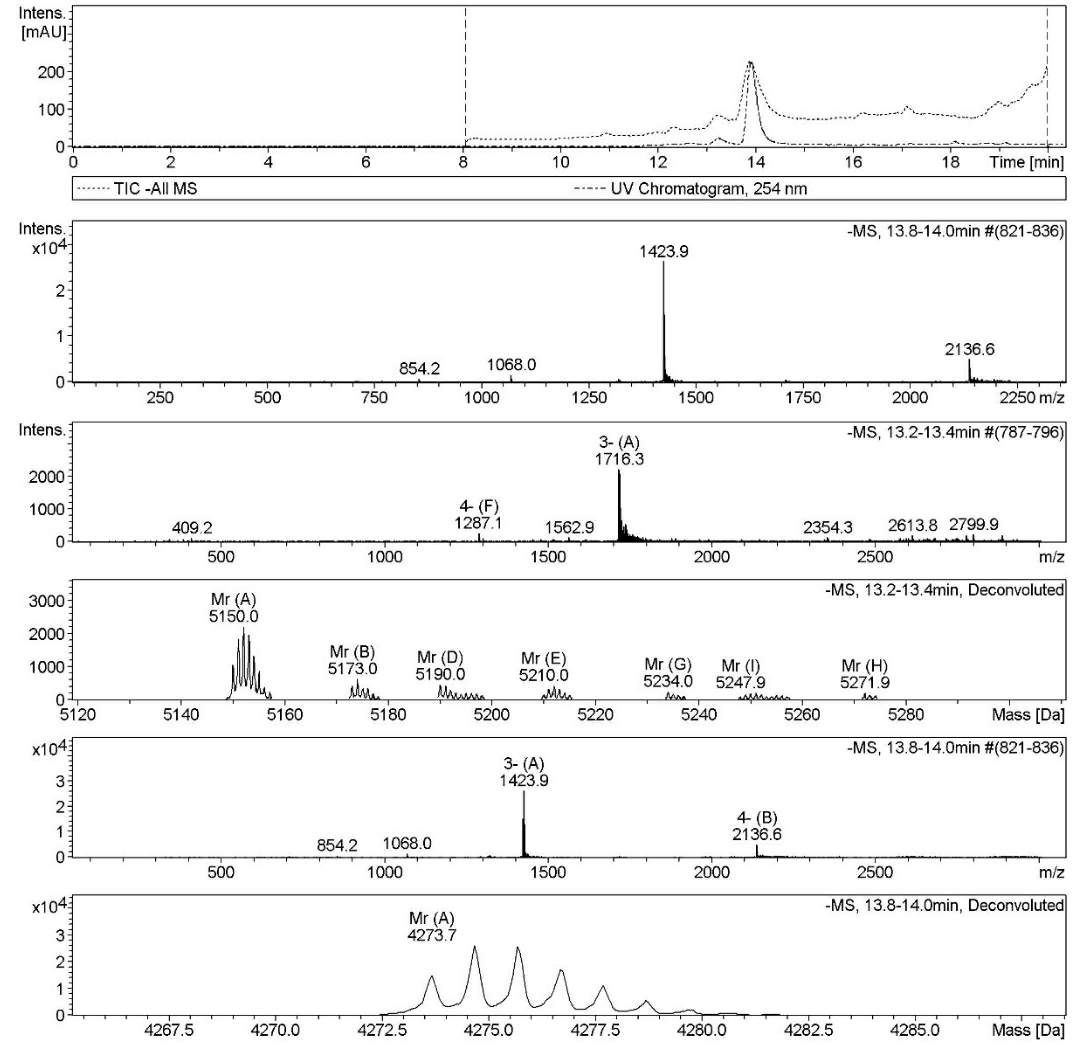

Bruker Compass DataAnalysis 4.0 printed: 19.10.2017 11:37:19 Page 1 of 1

ACAGATACGCA-BBQ650III

Acquisition Parameter

Source Type	ESI	Ion Polarity	Negative	Set Nebulizer	0.4 Bar
Focus	Active	Set Capillary	4500 V	Set Dry Heater	180 °C
Scan Begin	50 m/z	Set End Plate Offset	-500 V	Set Dry Gas	4.0 l/min
Scan End	3000 m/z	Set Collision Cell RF	400.0 Vpp	Set Divert Valve	Waste

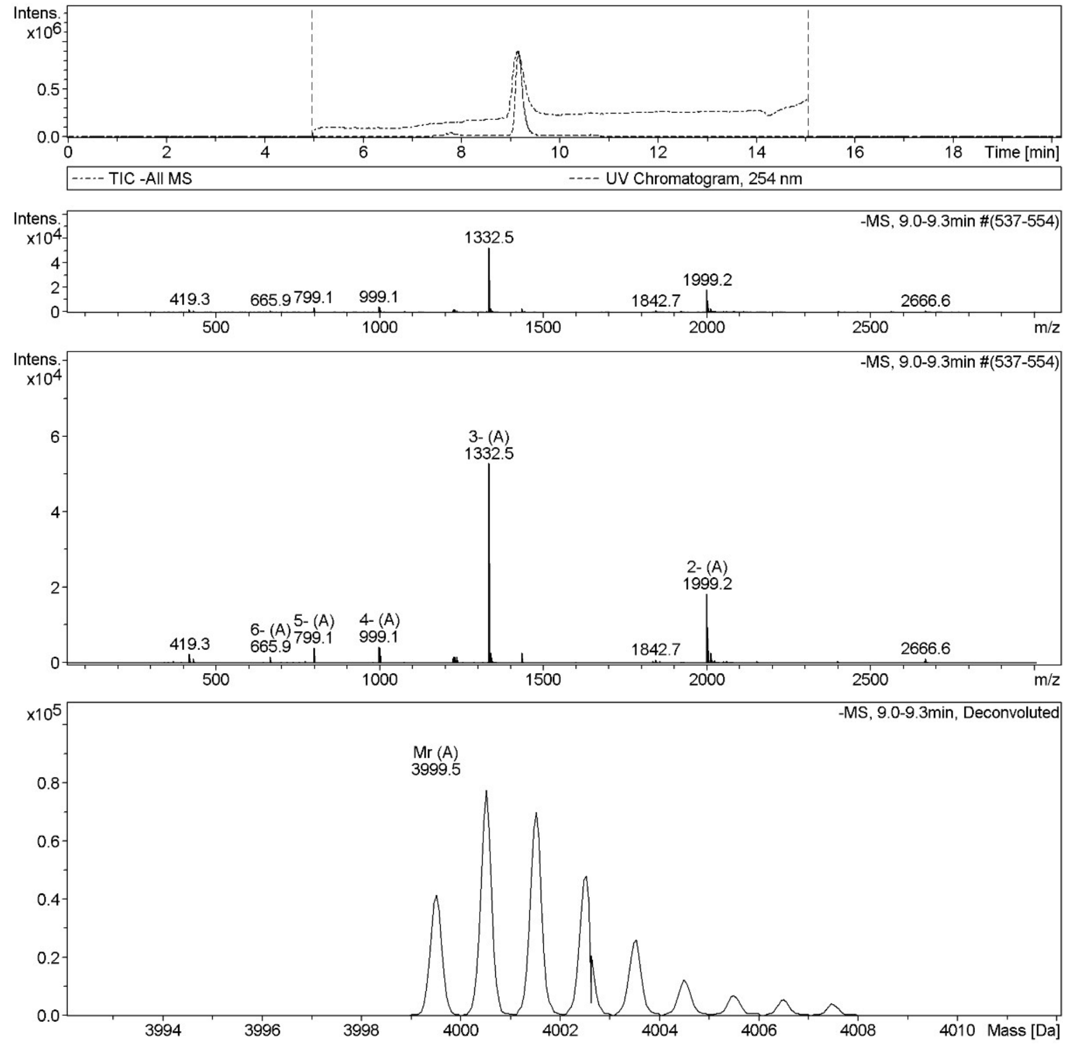

Bruker Compass DataAnalysis 4.0 printed: 13.01.2015 08:42:09 Page 1 of 1
